# A Stable Homoleptic Divinyl Tetrelene Series

**DOI:** 10.1002/chem.202100969

**Published:** 2021-05-14

**Authors:** Matthew M. D. Roy, Samuel R. Baird, Eike Dornsiepen, Lucas A. Paul, Linkun Miao, Michael J. Ferguson, Yuqiao Zhou, Inke Siewert, Eric Rivard

**Affiliations:** ^1^ Department of Chemistry University of Alberta 11227 Saskatchewan Dr. Edmonton Alberta T6G 2G2 Canada; ^2^ Universität Göttingen Institut für Anorganische Chemie Tammannstr. 4 37077 Göttingen Germany

**Keywords:** ligand design, main group, silylene, tetrelene, vinyl

## Abstract

The synthesis of the new bulky vinyllithium reagent (^Me^IPr=CH)Li, (^Me^IPr=[(MeCNDipp)_2_C]; Dipp=2,6‐*i*Pr_2_C_6_H_3_) is reported. This vinyllithium precursor was found to act as a general source of the anionic 2σ, 2π‐electron donor ligand [^Me^IPr=CH]^−^. Furthermore, a high‐yielding route to the degradation‐resistant Si^II^ precursor ^Me^IPr⋅SiBr_2_ is presented. The efficacy of (^Me^IPr=CH)Li in synthesis was demonstrated by the generation of a complete inorganic divinyltetrelene series (^Me^IPrCH)_2_E: (E=Si to Pb). (^Me^IPrCH)_2_Si: represents the first two‐coordinate acyclic silylene not bound by heteroatom donors, with dual electrophilic and nucleophilic character at the Si^II^ center noted. Cyclic voltammetry shows this electron‐rich silylene to be a potent reducing agent, rivalling the reducing power of the 19‐electron complex cobaltocene (Cp_2_Co).

## Graphical abstract

**Silylene? NHO Problem**! Access to a new lithiated anionic N‐heterocyclic olefin (NHO) yields a complete acyclic divinyltetrelene series.

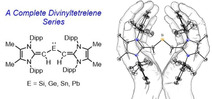

## Introduction

**Figure 1:**
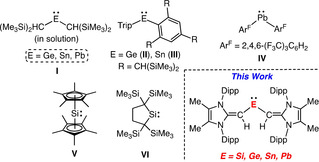
Alkyl‐ and aryl‐substituted tetrelenes; Trip=2,4,6‐*i*Pr_3_C_6_H_2_.

The seminal isolation of two‐coordinate acyclic dialkyl tetrelenes by Lappert and co‐workers in the 1970s (**I**, Figure 1),^[1]^ helped spur interest in the study of low‐coordinate main group compounds.^[2]^ Employment of more bulky aryl ligands kinetically inhibits dimerization in the solid state to afford monomeric germylenes (**II**), stannylenes (**III**) and plumbylenes (**IV**) (Figure 1).^[3]^ Renewed interest in tetrelenes stems from their uncovered transition‐metal like reactivity (e. g., H_2_ activation), and by their important role in main group catalysis.^[4]^ Strikingly absent from the two‐coordinate tetrelene family is a stable acyclic diorganosilylene.^[5]^ Notably, organosilylenes are postulated intermediates in the industrial “Direct Synthesis” of Me_2_SiCl_2_ and in the preparation of polysilanes [R_2_Si]_*n*_ via Wurtz coupling;[^6a^, ^6b^] moreover, organosilylenes (e. g., SiMe_2_) have been studied via matrix isolation.^[6c]^

Known diorganosilylenes are limited to Jutzi's coordinatively saturated silicocene Cp*_2_Si: (**V**; Cp*=C_5_Me_5_^−^)^[7]^ and Kira's cyclic silylene (**VI**) (Figure 1).^[5c]^ Two‐coordinate acyclic silylenes were first isolated in 2012[^5a^, ^5b^] with additional examples having since been reported;^[8]^ in all cases, stabilizing heteroatom donors are required. Herein we report the first complete divinyltetrelene series (Si−Pb) supported by the bulky vinylic donor [^Me^IPr=CH]^−^ (^Me^IPr=[(MeCNDipp)_2_C:]; Dipp=2,6‐*i*Pr_2_C_6_H_3_); this work required the development of the new reagent (^Me^IPr=CH)Li. We also introduce a high‐yielding route to a Si^II^ source that is less prone to degradation, leading to the isolation of the electron‐rich diorganosilylene (^Me^IPrCH)_2_Si:.

## Results and Discussion

This study arose from the desire to find a general method to install the bulky anionic *N*‐heterocyclic olefin (vinyl) ligand [^Me^IPr=CH]^−^ onto main group elements. Previously, this anionic ligand has been accessed via the *in situ* deprotonation of the *N*‐heterocyclic olefin (NHO)^[9]^ ^Me^IPr=CH_2_ within the coordination sphere of element halides. While this *in situ* route is viable in some cases,^[10]^ it is not universally successful. For example, we utilized this strategy to prepare the divinylgermylene (^Me^IPrCH)_2_Ge:,^[11]^ however attempts to coordinate Si^II^, Sn^II^ or Pb^II^ centers failed.

**Scheme 1:**
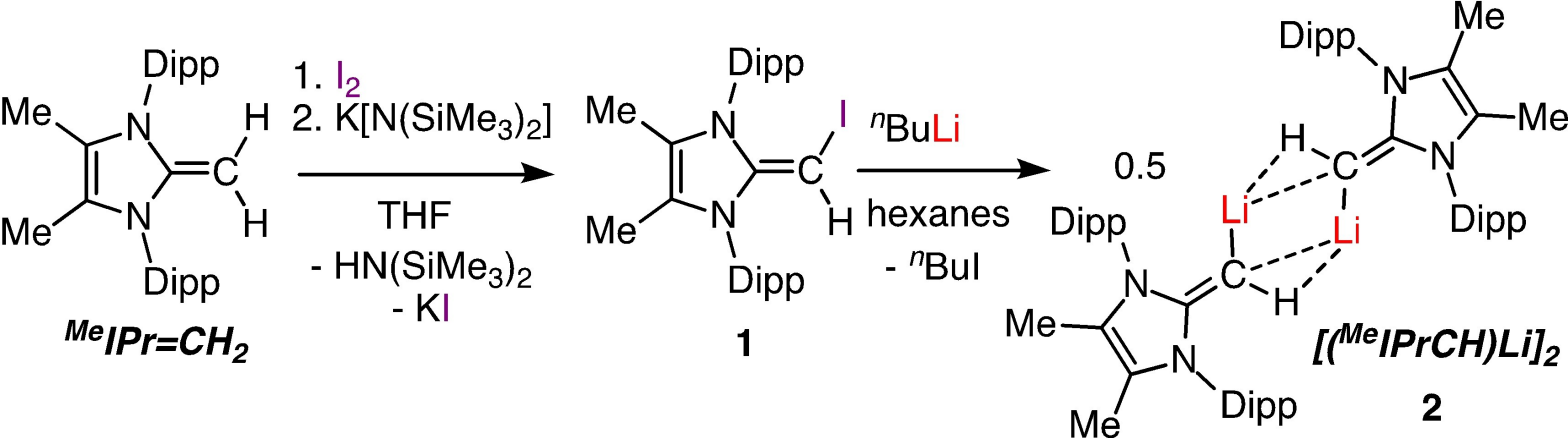
One‐pot iodination of ^Me^IPr=CH_2_ to form ^Me^IPr=CH(I) (**1**) and the subsequent lithiation of **1** to yield [(^Me^IPrCH)Li]_2_ (**2**).

To fully exploit the desirable 2σ, 2π‐electron donor capabilities of the bulky [^Me^IPr=CH]^−^ vinyl ligand,^[12]^ the lithiated reagent (^Me^IPr=CH)Li was targeted. As backbone ring deprotonation of the *N*‐heterocyclic olefin IPr=CH_2_ [IPr=(HCNDipp)_2_C:] occurs with alkyllithium reagents,^[13]^ we instead attempted to deprotonate the terminal =CH_2_ unit in the backbone methylated NHO ^Me^IPr=CH_2_ using *n*BuLi, *t*BuLi or *n*BuLi/TMEDA (TMEDA=tetramethylethylenediamine); however in each case no reaction transpired. As a result we moved to lithiation of the iodinated NHO ^Me^IPr=CH(I) (**1**) as a route to (^Me^IPr=CH)Li (Scheme 1).

When ^Me^IPr=CH_2_ was combined with I_2_ in THF, a bright yellow precipitate formed, tentatively assigned as the imidazolium salt [^Me^IPrCH_2_(I)]I.^[14]^ Upon subsequent addition of the strong amide base K[N(SiMe_3_)_2_], the precipitate was consumed and the target compound ^Me^IPr=CH(I) (**1**) was obtained as a yellow solid after its extraction into hexanes and isolation (Scheme 1). ^Me^IPr=CH(I) (**1**) crystallizes as a monomer in the solid state (Figure S25 in Supporting Information)^[15]^ with sp^2^‐character at the exocyclic carbon atom [C=C−I angle=128.1(3)°]. Thus far, our attempts to apply this iodination strategy to less bulky *N*‐heterocyclic olefins, such as the previously unreported ImMe_2_ *i*Pr_2_=CH_2_ [ImMe_2_ *i*Pr_2_=(MeCN*i*Pr)_2_C] and the related benzylated derivative [C_6_H_4_(N*i*Pr)_2_C=CH_2_] failed to yield clean products.

**Figure 2:**
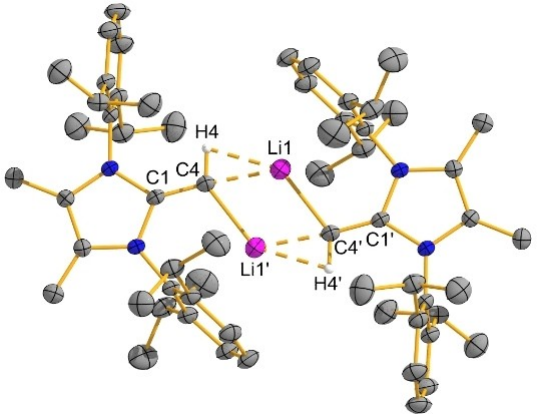
Molecular structure of [(^Me^IPrCH)Li]_2_ (**2**) with thermal ellipsoids plotted at a 30 % probability level. All hydrogen atoms (except the vinylic hydrogen atoms) were omitted for clarity. Selected bond lengths [Å] and angles [°]: C1‐C4 1.341(3), C4‐H4 0.94(3), C4‐Li1 2.027(5), C4‐Li1′ 2.117(5); C1‐C4‐Li1 164.2(3) C1‐C4‐Li1′ 111.9(2).

With compound **1** in hand, its successful lithiation with *n*BuLi transpired in hexanes to yield (^Me^IPr=CH)Li (**2**) as a bright orange solid in a 82 % yield (Scheme 1). Compound **2** crystallizes as a centrosymmetric dimer [(^Me^IPrCH)Li]_2_ (Figure 2) supported by core agostic (CH)−Li interactions [C4‐Li1: 2.027(5) Å], with the retention of considerable double bond character within the exocyclic olefin units [e. g., C1–C4: 1.341(3) Å]; for comparison, the neutral NHO ^Me^IPr=CH_2_ has a terminal C=C length of 1.3489(18) Å.^[9a]^ Diffusion‐ordered NMR spectroscopy (DOSY) revealed that the dimeric nature of **2** is retained in benzene.^[15]^ Solid **2** decomposes over a few days at room temperature, so it is advisable to store this species at −30 °C (where it is stable).

**Scheme 2:**
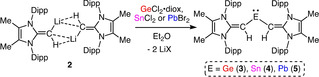
Formation of the divinylgermylene (**3**), divinylstannylene (**4**) and divinylplumbylene (**5**) from the vinyllithium precursor **2**.

To test whether [(^Me^IPrCH)Li]_2_ (**2**) would act as an effective source of the [^Me^IPr=CH]^−^ ligand, **2** was combined with Cl_2_Ge⋅dioxane in Et_2_O, which afforded the known deep‐red germylene (^Me^IPrCH)_2_Ge: (**3**) (Scheme 2).^[11]^ Previous attempts to form a tin‐containing divinyltetrelene were unsuccessful, however combining **2** with SnCl_2_ afforded (^Me^IPrCH)_2_Sn: (**4**) as pink‐red crystals in a 32 % isolated yield (Scheme 2).^[16]^

**Figure 3:**
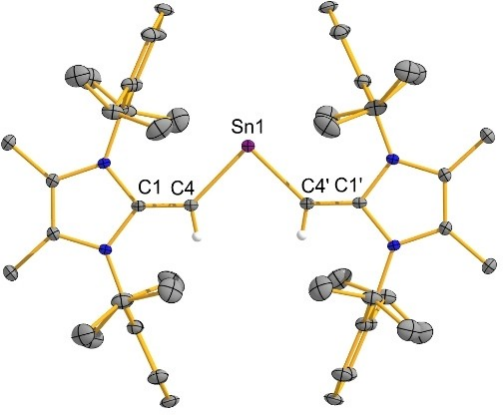
Molecular structure of (^Me^IPrCH)_2_Sn: (**4**) with thermal ellipsoids plotted at a 30 % probability level. All hydrogen atoms (except the vinylic hydrogen atoms) were omitted for clarity. Selected bond lengths [Å] and angles [°]: C1‐C4 1.352(5), C4‐Sn1 2.109(3); C4‐Sn1‐C4′ 90.83(14).

The X‐ray crystallographic structure of **4** is shown in Figure 3 and the vinylic C=C bonds [1.352(5) Å] are the same length (within error) as in free ^Me^IPr=CH_2_ [1.3489(18) Å].^[9a]^ Our supporting computations reveal that there is minimal π‐overlap between the polarized C=C π‐units and the relatively diffuse empty 5p orbital on Sn.^[15]^ The C−Sn−C bond angle in **4** adopts nearly a right angle [90.83(14)°], consistent with predominantly s‐character of the Sn lone pair. The ^119^Sn{^1^H} spectrum of **4** displays a broad singlet resonance at 1162 ppm, which is significantly upfield‐shifted when compared to Aldridge's related vinylated stannylene Sn{(Ph)C=C(H)B(NDippCH)_2_}_2_ (1730 ppm).^[2f]^

The divinylplumbylene (^Me^IPrCH)_2_Pb: (**5**) was synthesized from [(^Me^IPrCH)Li]_2_ (**2**) and PbBr_2_ in Et_2_O and isolated as deep‐blue crystals in a 49 % yield (Scheme 2). The crystallographically determined structure of **5** (Figure S27)^[15]^ is very similar to that of the divinylstannylene **4**, with a narrow central C−Pb−C angle of 88.6(2)°. This angle is more contracted than the corresponding angle of 94.5(1)° in the first known diarylplumbylene **IV** (see Figure 1).^[3c]^ Additionally, compound **5** exhibits a downfield‐shifted ^207^Pb{^1^H} resonance of 5449 ppm when compared to that of **IV** (4878 ppm).^[3c]^

**Scheme 3:**
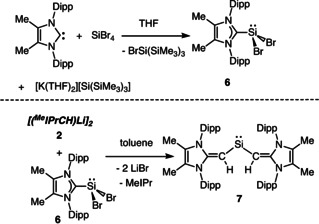
Formation of the Si^II^ precursor **6** (top) and the divinylsilylene (^Me^IPrCH)_2_Si: (**7**) (bottom).

Finding a suitable path to the Si^II^ analogue of the tetrelenes **3–5** proved to be a non‐trivial task. When [(^Me^IPrCH)Li]_2_ (**2**) was mixed with either Roesky's (IPr⋅SiCl_2_) or Filippou's (IPr⋅SiBr_2_) Si^II^ precursors,^[17]^ undesired carbene ligand C−H deprotonation by **2** and regeneration of ^Me^IPr=CH_2_ was observed. In order to circumvent this degradation pathway, the backbone methylated Si^II^ adduct ^Me^IPr⋅SiBr_2_ (**6**)^[18]^ was prepared using a new one‐pot procedure. Motivated by the prior use of the hypersilyl salt [K(THF)_2_][Si(SiMe_3_)_3_] to reduce Si^IV^ to Si^II^ centers,[^8a^, ^8c^, ^8f^] combining this [Si(SiMe_3_)_3_]^−^ reagent with ^Me^IPr and then SiBr_4_ cleanly afforded the corresponding Si^II^ dibromide adduct ^Me^IPr⋅SiBr_2_ (**6**) in a 64 % yield (Scheme 3). The hypersilyl bromide byproduct BrSi(SiMe_3_)_3_ could be easily removed by washing the product with cold (−30 °C) hexanes.

Combining ^Me^IPr⋅SiBr_2_ (**6**) with [(^Me^IPrCH)Li]_2_ (**2**) in toluene led to the immediate formation of a deep‐yellow/brown mixture. Work‐up, including crystallization of the soluble species from hexanes at −30 °C gave deep‐yellow crystals of the target divinylsilylene (^Me^IPrCH)_2_Si: (**7**) in a 27 % yield (Scheme 3). The most salient spectroscopic feature of **7** is its ^29^Si NMR chemical shift of 272 ppm (in C_6_D_6_); this value is quite shielded with respect to the recently reported vinyl silylsilylene (^Me^IPrCH)Si{Si(SiMe_3_)_3_} (433 ppm)^[8f]^ and is comparable to known amido‐silylenes.[^8b^, ^8c^]

**Figure 4:**
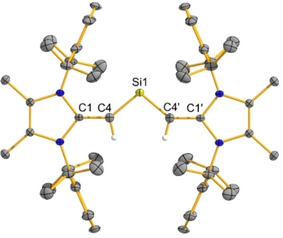
Molecular structure of (^Me^IPrCH)_2_Si: (**7**) with thermal ellipsoids plotted at a 30 % probability level. All hydrogen atoms (except the vinylic/olefinic hydrogen atoms) were omitted for clarity. Selected bond lengths [Å] and angles [°]: C1‐C4 1.375(2), C4‐Si1 1.7620(14); C1‐C4‐Si1 140.29(4), C4‐Si1‐C4′ 100.58(8).

As shown in Figure 4, the C=C bond length in (^Me^IPrCH)_2_Si: (**7**) [C1‐C4: 1.375(2) Å] is elongated relative to ^Me^IPr=CH_2_ [1.3489(18) Å],^[9a]^ consistent with π‐donation from the vinylic C=C bonds to an adjacent silicon‐based p‐orbital in **7**. The presence of delocalized C−Si−C π‐bonding was located as the HOMO‐2 by computations (B3LYP/cc‐pVDZ level).^[15]^ The core C−Si−C angle in **7** [100.58(8)°] is consistent with mixed s/p character of the Si lone pair, as is expected for this lighter ER_2_ congener.

While **7** is highly sensitive to moisture, it is stable in benzene solution for weeks under N_2_ and only decomposes in the solid state upon heating to 155 °C. The reactivity of silylenes is commonly probed by addition of H_2_.^[5a]^ However, thus far, we have found **7** to be stable under an atmosphere of dihydrogen. This observation agrees with the high degree of s‐orbital character (71 %) of the lone pair according to NBO analysis and the correspondingly large HOMO‐LUMO and singlet‐triplet energy gaps (ΔE_H‐L_: 73.3 kcal mol^−1^, ΔE_S‐T_: 37.6 kcal mol^−1^).^[15]^ The singlet‐triplet gap is reminiscent of a previously reported diaminosilylene (TBoN)_2_Si [TBoN={(HCNDipp)_2_B}N(SiMe_3_)] (ΔE_S‐T_: 37.8 kcal mol^−1^), which does not react with H_2_, even at elevated temperatures (60 °C) and pressures (40 atm).^[8b]^

**Figure 5:**
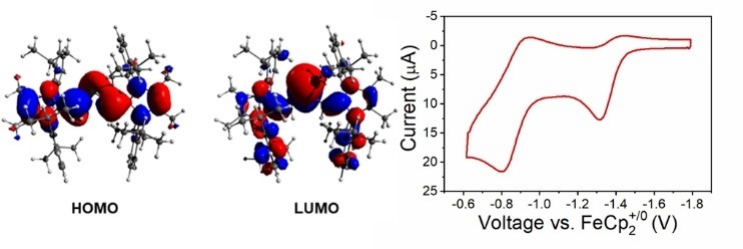
(left) Frontier molecular orbitals of the optimized structure of (^Me^IPrCH)_2_Si: (**7**) at the B3LYP/cc‐pVDZ level of theory. (right) CV data of **7** showing two oxidation events at a rapid scan rate of 20 Vs^−1^ in benzene (0.2 M [*n*Hex_4_N][B(C_6_F_5_)_4_] as an electrolyte).

Electrochemical measurements on **7** in benzene at room temperature (with 0.2 M [*n*Hex_4_N][B(C_6_F_5_)_4_] as an electrolyte) revealed three irreversible oxidation processes at peak potentials of *E*_p,a,1_=−1.35 V, *E*_p,a,2_=−0.84 V, and *E*_p,a,3_=−0.76 V vs. FeCp_2_^+/0^, at scan rates of 0.1 V s^−1^ (Figures S28 and S29).^[15]^ With increasing scan rates (up to 20 Vs^−1^; Figure 5) the first two oxidation events become more reversible and half wave potentials E_1/2_ of −1.35 V for the initial process and of −0.84 V for the second process could be estimated. The third oxidation event vanishes with increasing scan rates (>2 V s^−1^), which suggests that it belongs to a species formed in the quick follow‐up reaction after initial oxidation of **7**. Overall, the CV data is consistent with **7** undergoing two successive one‐electron processes, forming a formal Si^IV^ compound via a short‐lived radical species. Based on the half peak potential at −1.35 V we can say that **7** is a potentially strong reducing agent in benzene, with a redox potential similar to that of the 19‐electron reductant cobaltocene, Cp_2_Co (compare to −1.33 vs. FeCp_2_^+/0^ in CH_2_Cl_2_).^[19]^

**Figure 6:**
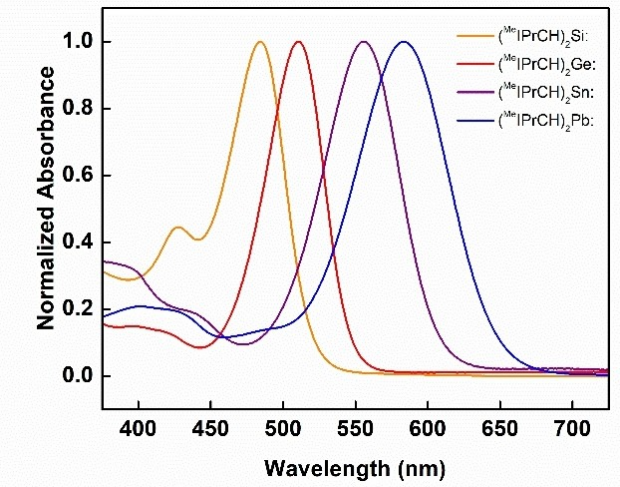
UV‐vis spectra for the divinyltetrelene series (^Me^IPrCH)_2_E: (E=Si, Ge, Sn and Pb; **3–5** and **7**) in hexanes.

The deep colors of the divinyltetrelenes **3–5** and **7** prompted further computational and spectroscopic investigations. UV‐vis spectroscopy in hexanes revealed a progressive red‐shift in λ_max_ on going from Si to Pb (λ_max_=484, 511, 557 and 583 nm, respectively; Figure 6).^[15]^ TD‐DFT at the B3LYP/cc‐pVDZ level shows that the spectra are dominated by symmetry allowed HOMO→LUMO transitions, with the HOMO primarily consisting of a ligand‐based π‐orbital located on the polarized vinyl C=C bonds of the ligands, and the LUMO being predominantly E(p) in character with decreasing C−E π* contributions upon going down Group 14.^[15]^ The observed red‐shift in λ_max_ in the ER_2_ series from Si to Pb can be partially explained by the less effective π‐overlap between the vinyl ligands and the tetrelene upon descending the group. For the heavier elements, the HOMO becomes destabilized while the LUMO is stabilized, thus leading to a narrowing of the HOMO‐LUMO gap, as reflected in the UV‐vis data.

**Figure 7:**
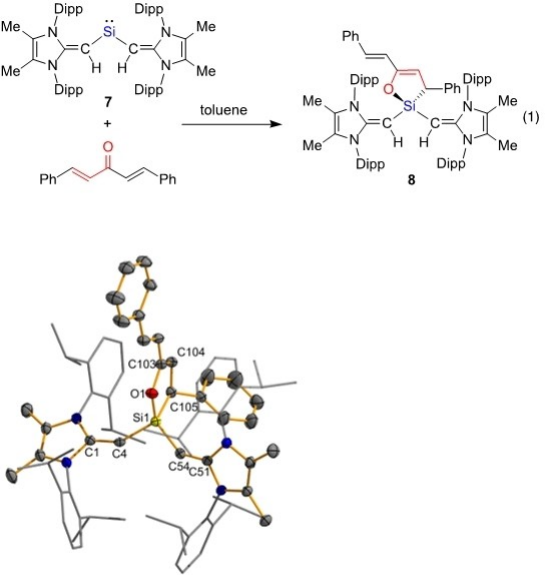
Molecular structure of (^Me^IPrCH)_2_Si(dba) (**8**) with thermal ellipsoids plotted at a 30 % probability level. All hydrogen atoms are omitted for clarity, while all Dipp groups are shown in wireframe. Selected bonds lengths [Å] and angles [°]: Si1‐C4 1.805(4), C1‐C4 1.394(5), Si1‐O1 1.700(3), Si1‐C105 1.926(4); C4‐Si1‐C54 100.57(16), O1‐Si1‐C105 93.36(15).

Initial attempts to generate silylene d^10^ metal complexes by combining **7** with either Ni(COD)_2_ (COD=1,5‐cyclooctadiene), Pd(P*t*Bu_3_)_2_ or Pt(P*t*Bu_3_)_2_ in benzene at 55 °C (for 3 days) led to no discernable reaction. Treatment of **7** with one equiv. of [Pd_3_(dba)_2_] (dba=dibenzylideneacetone) yielded a small batch of yellow crystals, which were identified as the silane (^Me^IPrCH)_2_Si(dba) (**8**) (Figure 7); compound **8** was then obtained in an independent reaction between **7** and dba (Equation 1 in Figure 7). This formal [4+1] cycloaddition process demonstrates the dual electrophilic/nucleophilic character of the Si^II^ center in **7**.

## Conclusion

We have gained access to the novel lithiated vinyl dimer [(^Me^IPrCH)Li]_2_ (**2**), a universal source of the potential 4‐electron donor [^Me^IPr=CH]^−^. From **2**, the complete divinyl tetrelene series (^Me^IPrCH)_2_E: was obtained (E=Si−Pb), with isolation of the Si^II^ congener necessitating the use of the structurally protected Si^II^ precursor ^Me^IPr⋅SiBr_2_ (**6**). ^Me^IPr⋅SiBr_2_ should be of great value to those looking to further develop low‐oxidation state Si chemistry via nucleophilic halide replacement. Furthermore, electron‐rich (^Me^IPrCH)_2_Si: (**7**) represents the first example of an acyclic two‐coordinate diorganosilylene. Future work will involve exploring new bonding modes supported by the bulky vinyl substituent [^Me^IPr=CH]^−^, including the exploration of catalysis mediated by low‐coordinate inorganic elements.

## Experimental Section

**General**: All reactions were performed in an inert atmosphere glovebox (manufactured by Innovative Technology, Inc.) or via a Schlenk line, both under nitrogen. Solvents were dried using a Grubbs‐type solvent purification system^[20]^ supplied by Innovative Technologies, Inc., degassed (freeze‐pump‐thaw method), and stored under an atmosphere of nitrogen prior to use. Cl_2_Ge⋅dioxane, SnCl_2_, PbBr_2_, K[N(SiMe_3_)_2_], *n*BuLi (2.5 M solution in hexanes), *i*PrI and I_2_ were purchased from Aldrich and used as received. SiBr_4_ was purchased from Alfa Aesar and used as received. ^Me^IPr,^[21]^ ^Me^IPr=CH_2_,^[9a]^ (^Me^IPr=[(MeCNDipp_2_C]; Dipp=2,6‐*i*Pr_2_C_6_H_3_), [K(THF)_2_][Si(SiMe_3_)_3_],^[22]^ 2,4,5‐trimethylimidazole,^[23]^ and dibenzylideneacetone^[24]^ were prepared according to literature procedures. ^1^H, ^13^C{^1^H}, ^29^Si, ^119^Sn and ^207^Pb NMR spectra were recorded on 400, 500, 600 or 700 MHz Varian Inova instruments and were referenced externally to SiMe_4_ (^1^H, ^13^C{^1^H}, ^29^Si), a 9.7 M solution of LiCl in D_2_O (^7^Li), SnMe_4_ (^119^Sn) or Me_4_Pb (^207^Pb). Elemental analyses were performed by the Analytical and Instrumentation Laboratory at the University of Alberta. Melting points were measured in sealed glass capillaries under nitrogen by using a MelTemp apparatus and are uncorrected. UV‐Visible spectroscopic measurements were carried out with a Varian Carry 300 Scan spectrophotometer. Diffusion‐ordered spectroscopy (DOSY) experiments were performed on a Varian 400 MHz instrument equipped with a Z‐gradient broadband probe capable of outputting 61.6 G cm^−1^ of gradient strength. All measurements were carried out non‐spinning samples and at a calibrated temperature of 27 °C using the Oneshot45 pulse sequence.[^25^, ^26^] For all DOSY experiments a spectral window of 6 kHz was used with a 3 s acquisition time and a 2 s S4 relaxation delay with 8 scans for each gradient increment. Pulse widths and gradient strengths were optimized for each sample. A diffusion delay of 100 ms and a diffusion gradient length of 2 ms was used. Gradient strengths of 1.9 to 14.1 G cm^−1^ incremented in 20 steps were used. The spectra were Fourier transformed and baseline corrected prior to discrete processing, fitting the data to a double exponential fit and applying corrections for non‐uniform gradients.^[27]^ The diffusion dimension was zero filled to 1024 data points and the directly detected dimension was zero filled to 128 K data points.

**X‐ray crystallography**: Crystals for X‐ray diffraction studies were removed from a vial (in a glovebox) and immediately coated with a thin layer of hydrocarbon oil (Paratone‐N). A suitable crystal was then mounted on a glass fibre and quickly placed in a low temperature stream of nitrogen on the X‐ray diffractometer.^[28]^ All data were collected using a Bruker APEX II CCD detector/D8 or PLATFORM diffractometer using Mo_K*α*_ or Cu_K*α*_ radiation, with the crystals cooled to −80 °C or −100 °C. The data were corrected for absorption through Gaussian integration from the indexing of the crystal faces. Crystal structures were solved using intrinsic phasing (*SHELXT*)^[29]^ and refined using *SHELXL‐2014*.^[30]^ The assignment of hydrogen atom positions was based on the sp^2^ or sp^3^ hybridization geometries of their attached carbon atoms and were given thermal parameters 20 % greater than those of their parent atoms.

**Electrochemical measurements**: The cell was prepared in a nitrogen‐filled glovebox with dry benzene as the solvent. All CV measurements were conducted with a common three electrode set‐up consisting of a glassy carbon (GC) working electrode, a platinum wire counter electrode, and a silver wire as pseudo reference electrode. 0.2 M solution of [*n*Hex_4_N][B(C_6_F_5_)_4_] in benzene was used as the electrolyte. A Gamry Reference 600+ potentiostat was used for the measurements. i*R* compensation was performed by the positive feedback method, which is implemented in the PHE200 software. All data were referenced internally vs. CoCp*_2_. CoCp*_2_ was measured independently vs. FeCp_2_ under otherwise identical conditions. The CoCp*_2_^+^|CoCp*_2_ redox couple was determined to be −2.050 V vs. the FeCp_2_^+^|FeCp_2_ redox couple in benzene with a standard deviation of 5 mV (6 measurements). Accordingly, this value has been used to reference the CV data of **7** vs. FeCp_2_^+^|FeCp_2_.

**Computational methods**: All computations were carried out using the Gaussian 16 software package.^[31]^ Experimentally determined molecular structures were used as input structures and optimized using the B3LYP^[32]^ functional and the cc‐pVDZ^[33]^ basis set (for C, H, N, Si and Ge atoms) in the gas phase. The cc‐pVDZ‐PP^[34]^ basis set was applied to Sn and Pb atoms which includes an effective core potential (ECP) accounting for 28 electrons (Sn) or 60 electrons (Pb). All structures were confirmed to be minima on the potential energy surface using frequency analysis. The vertical excitation energies of the first ten singlet and triplet states were predicted by TD‐DFT computations using the respective optimized gas‐phase singlet ground‐state (S_0_) geometries. Natural bond orbital (NBO) analysis was conducted using NBO 3.1.^[35]^

**Synthesis of** ^**Me**^ **IPr=CH(I) (1)**: A solution of I_2_ in 20 mL THF (1.153 g, 4.543 mmol) was added via syringe to a solution of ^Me^IPrCH_2_ (1.997 g, 4.637 mmol) in 150 mL of THF in a Schlenk flask. Upon addition of I_2_, a flocculant, bright‐yellow precipitate began to form. Once the addition was complete, the resulting mixture was stirred for an additional 140 minutes. *From this point on, all manipulations were conducted in the absence of ambient light*. A solution of K[N(SiMe_3_)_2_] (0.920 g, 4.61 mmol) in THF was transferred via cannula to the yellow mixture. Upon addition of the amide base, the yellow precipitate was slowly consumed to form a brown slurry. This mixture was stirred for 70 minutes and the volatiles were subsequently removed *in vacuo*. The solid residue was extracted with 100 mL of hexanes and filtered through a frit packed with a ca. 1 cm plug of diatomaceous earth. The resulting dark yellow filtrate was concentrated to 20 mL and placed in a −30 °C freezer for 16 hours, which afforded bright yellow crystals of ^Me^IPr=CH(I) (1.127 g). The mother liquor was then concentrated to half its original volume and placed in a −30 °C freezer for 16 hours, yielding a second crop of crystals (0.326 g; combined yield: 1.453 g, 57 %). Yellow crystals that were of suitable quality for X‐ray diffraction analysis were obtained by dissolving **1** in a minimal amount of hexanes and storing the solution at −30 °C for one week. ^1^H NMR (400 MHz, C_6_D_6_): δ 7.32 (t, 1H, ^3^ *J*_HH_=7.6 Hz, *p*‐Ar*H*), 7.22‐7.17 (m, 3H, *p*‐ and *m*‐Ar*H*), 7.10 (d, 2H, ^3^ *J*_HH_=7.6 Hz, *m*‐Ar*H*), 3.24 (sept, 2H, ^3^ *J*_HH_=6.8 Hz, C*H*(CH_3_)_2_), 3.13 (sept, 2H, ^3^ *J*_HH_=6.8 Hz, C*H*(CH_3_)_2_), 2.25 (s, 1H, C*H*I), 1.59 (d, 6H, ^3^ *J*_HH_=6.8 Hz, CH(C*H*_3_)_2_), 1.52 (s, 3H, NCC*H*_3_), 1.46 (s, 3H, NCC*H*_3_), 1.34 (d, 6H, ^3^ *J*_HH_=6.8 Hz, CH(C*H*_3_)_2_), 1.19 (d, 6H, ^3^ *J*_HH_=6.8 Hz, CH(C*H*_3_)_2_), 1.16 (d, 6H, ^3^ *J*_HH_=6.8 Hz, CH(C*H*_3_)_2_). ^13^C{^1^H} NMR (100 MHz, C_6_D_6_): δ 149.3 (Ar*C*), 149.2 (Ar*C*), 145.6 (N*C*N), 133.0 (Ar*C*), 132.5 (Ar*C*), 129.8 (Ar*C*), 129.7 (Ar*C*), 124.7 (Ar*C*), 123.8 (Ar*C*), 117.0 (N*C*‐CH_3_), 116.6 (N*C*‐CH_3_), 29.1 (*C*H(CH_3_)_2_), 28.9 (*C*H(CH_3_)_2_), 24.8 (CH(*C*H_3_)_2_), 24.3 (CH(*C*H_3_)_2_), 23.9 (CH(*C*H_3_)_2_), 9.4 (NC‐*C*H_3_), 9.3 (NC‐*C*H_3_), −7.1 (*C*HI). Anal. Calcd. for C_30_H_41_IN_2_: C 64.74, H 7.43, N 5.03; Found: C 64.89, H 7.45, N 4.86. M.p. 150 °C (decomp.).

**Synthesis of [(^Me^IPrCH)Li]_2_ (2)**: A 2.5 M solution of ^n^BuLi in hexanes (208 μL, 0.519 mmol) was added to a yellow solution of ^Me^IPr=CH(I) (0.289 g, 0.519 mmol) in 4 mL of hexanes. After 2 minutes of stirring, the solution began to turn a bright orange/red color. After 20 minutes of stirring, an orange/red solid began to precipitate from solution. The mixture was placed in a −30 °C freezer for 16 hours, then the mother liquor was decanted from the resulting precipitate (and discarded) and the volatiles were removed *in vacuo* yielding [(^Me^IPrCH)Li]_2_ (**2**) as a bright orange/red solid (0.185 g, 82 %). Crystals suitable for X‐ray crystallographic analysis (orange‐red) were obtained by storing a hexanes solution of **2** in a −30 °C freezer for 10 days. *Compound **2** slowly decomposes at room temperature, even when stored in an inert atmosphere in the solid state. As such, batches of **2** were always stored as a solid at* −*30 °C in a glovebox*. ^1^H NMR (C_6_D_6_, 400 MHz): δ 7.39 (t, 2H, ^3^ *J*_HH_=8.0 Hz, *p*‐Ar*H*), 7.32 (d, 4H, ^3^ *J*_HH_=8.0 Hz, *m*‐Ar*H*), 7.04 (d, 4H, ^3^ *J*_HH_=8.0 Hz, *m*‐Ar*H*), 6.87 (t, 2H, ^3^ *J*_HH_=8.0 Hz, *p*‐Ar*H*), 3.40‐3.13 (m, 8H, C*H*(CH_3_)_2_), 1.66 (s, 6H, NC‐C*H*_3_), 1.64 (s, 6H, NC‐C*H*_3_), 1.39 (d, 12H, ^3^ *J*_HH_=7.0 Hz, CH(C*H*_3_)_2_), 1.31 (d, 12H, ^3^ *J*_HH_=7.0 Hz, CH(C*H*_3_)_2_), 1.30 (d, 12H, ^3^ *J*_HH_=7.0 Hz, CH(C*H*_3_)_2_), 1.10 (d, 12H, ^3^ *J*_HH_=7.0 Hz, CH(C*H*_3_)_2_), 0.87 (broad s, 2H, C*H*Li). ^13^C{^1^H} NMR (C_6_D_6_, 176 MHz): δ 159.0 (N*C*N), 150.5 (Ar*C*), 150.4 (Ar*C*), 136.6 (Ar*C*), 136.0 (Ar*C*), 129.4 (Ar*C*), 128.3 (Ar*C*), 126.0 (Ar*C*), 123.7 (Ar*C*), 115.6 (N*C*CH_3_), 113.6 (N*C*CH_3_), 69.7 (broad, C=*C*Li), 28.7 (*C*H(CH_3_)_2_), 28.5 (*C*H(CH_3_)_2_), 25.4 (CH(*C*H_3_)_2_), 24.5 (CH(*C*H_3_)_2_), 24.3 (CH(*C*H_3_)_2_), 23.9 (CH(*C*H_3_)_2_), 10.3 (NC*C*H_3_), 10.1 (NC*C*H_3_). ^7^Li{^1^H} NMR (C_6_D_6_, 194 MHz): δ 1.0 (s). Anal. Calcd. for C_60_H_82_N_4_Li_2_: C 82.53, H 9.47, N 6.42. Found: C 80.26, H 9.25, N 6.11. M.p. 188–190 °C.

**Synthesis of (^Me^IPrCH)_2_Ge: (3)**: A solution of [(^Me^IPrCH)Li]_2_ (0.067 g, 0.077 mmol) in 4 mL of Et_2_O was added to a slurry of Cl_2_Ge⋅dioxane (0.018 g, 0.077 mmol) in 1 mL of Et_2_O. After stirring for 1 minute, the resulting mixture had turned deep red in color. After stirring for an additional 2 hours, the volatiles were removed *in vacuo*, the residue extracted with 5 mL of hexanes and filtered. The filtrate was concentrated to a volume of 2 mL and placed in a −30 °C freezer for one week. The mother liquor was decanted away from the resulting deep orange/red crystals of **3** and the crystals dried (0.035 g, 49 %). ^1^H and ^13^C{^1^H} data for **3** matched those found in the literature.^[11]^

**Synthesis of (^Me^IPrCH)_2_Sn: (4)**: A solution of [(^Me^IPrCH)Li]_2_ (0.043 g, 0.049 mmol) in 4 mL of Et_2_O was added to a slurry of SnCl_2_ (0.010 g, 0.053 mmol) in 1 mL of Et_2_O. After stirring for 1 minute, the resulting mixture had turned a deep violet in color. After stirring for an additional 30 minutes, the volatiles were removed *in vacuo*, the residue extracted with 4 mL of hexanes and filtered. The filtrate was concentrated to a volume of 2 mL and placed in a −30 °C freezer for one week. A few crystals were removed for X‐ray crystallographic analysis. The mother liquor was decanted from the bulk crystals and the crystals dried *in vacuo* affording (^Me^IPrCH)_2_Sn: (**4**) as a deep red‐pink crystalline solid (0.015 g, 32 %). ^1^H NMR (C_6_D_6_, 700 MHz): δ 7.33 (t, 2H, ^3^ *J*_HH_=8.0 Hz, *p*‐Ar*H*), 7.22 (t, 2H, ^3^ *J*_HH_=8.0 Hz, *p*‐Ar*H*), 7.15 (d, 4H, ^3^ *J*_HH_=8.0 Hz, *m*‐Ar*H*), 7.14 (d, 4H, ^3^ *J*_HH_=8.0 Hz, *m*‐Ar*H*), 5.19 (s, 2H, satellites: ^2^ *J*_HSn_=65.0 Hz, C*H*Sn), 3.20 (sept, 4H, ^3^ *J*_HH_=7.0 Hz, C*H*(CH_3_)_2_), 3.04 (sept, 4H, ^3^ *J*_HH_=7.0 Hz, C*H*(CH_3_)_2_), 1.59 (s, 6H, NC‐C*H*_3_), 1.58 (s, 6H, NC‐C*H*_3_), 1.34 (d, 12H, ^3^ *J*_HH_=7.0 Hz, CH(C*H*_3_)_2_), 1.21 (d, 12H, ^3^ *J*_HH_=7.0 Hz, CH(C*H*_3_)_2_), 1.17 (d, 12H, ^3^ *J*_HH_=7.0 Hz, CH(C*H*_3_)_2_), 1.14 (d, 12H, ^3^ *J*_HH_=7.0 Hz, CH(C*H*_3_)_2_). ^13^C{^1^H} NMR (C_6_D_6_, 176 MHz): δ 157.7 (N*C*N), 149.0 (Ar*C*), 147.0 (Ar*C*), 135.0 (Ar*C*), 133.4 (Ar*C*), 129.4 (Ar*C*), 128.8 (Ar*C*), 126.6 (Ar*C*), 125.5 (Ar*C*), 124.1 (Ar*C*), 117.6 (N*C*CH_3_), 116.7 (N*C*CH_3_), 49.1 (C=*C*H), 28.8 (*C*H(CH_3_)_2_), 28.7 (*C*H(CH_3_)_2_), 25.0 (CH(*C*H_3_)_2_), 24.9 (CH(*C*H_3_)_2_), 24.2 (CH(*C*H_3_)_2_), 24.0 (CH(*C*H_3_)_2_), 9.8 (NC*C*H_3_), 9.5 (NC*C*H_3_). ^119^Sn{^1^H} NMR ([D_8_]toluene, 149 MHz, −20 °C): δ 1162 (broad s). Anal. Calcd. for C_60_H_82_N_4_Sn: C 73.68, H 8.45, N 5.73. Found: C 72.62, H 8.25, N 5.22. M.p. 185 °C (decomp.). UV‐Vis: λ_max_=557 nm (ϵ=2200 M^−1^cm^−1^).

**Synthesis of (^Me^IPrCH)_2_Pb: (5)**: A solution of [(^Me^IPrCH)Li]_2_ (0.067 g, 0.076 mmol) in 4 mL of Et_2_O was added to a slurry of PbBr_2_ (0.033 g, 0.090 mmol) in 1 mL of Et_2_O. After stirring for 1 minute, the resulting mixture had turned a deep blue color. After stirring for an additional 20 minutes, the volatiles were removed *in vacuo*, the residue extracted with 4 mL of hexanes and filtered. The filtrate was concentrated to 2 mL and placed in a −30 °C freezer for one week. A few crystals were removed for X‐ray crystallographic analysis. The mother liquor was decanted from the bulk crystals and the crystals were dried *in vacuo* affording (^Me^IPrCH)_2_Pb: (**5**) as a deep blue crystalline solid (0.047 g, 49 %). ^1^H NMR (C_6_D_6_, 700 MHz): δ 7.35 (t, 2H, ^3^ *J*_HH_=8.0 Hz, *p*‐Ar*H*), 7.24 (s, 2H, C*H*Pb), 7.20 (t, 2H, ^3^ *J*_HH_=8.0 Hz, *p*‐Ar*H*), 7.17 (d, 4H, ^3^ *J*_HH_=8.0 Hz, *m*‐Ar*H*), 7.14 (d, 4H, ^3^ *J*_HH_=8.0 Hz, *m*‐Ar*H*), 3.24 (sept, 4H, ^3^ *J*_HH_=7.0 Hz, C*H*(CH_3_)_2_), 3.08 (sept, 4H, ^3^ *J*_HH_=7.0 Hz, C*H*(CH_3_)_2_), 1.61 (s, 12H, NC‐C*H*_3_), 1.27 (d, 12H, ^3^ *J*_HH_=7.0 Hz, CH(C*H*_3_)_2_), 1.22 (d, 12H, ^3^ *J*_HH_=7.0 Hz, CH(C*H*_3_)_2_), 1.16 (d, 12H, ^3^ *J*_HH_=7.0 Hz, CH(C*H*_3_)_2_), 1.15 (d, 12H, ^3^ *J*_HH_=7.0 Hz, CH(C*H*_3_)_2_). ^13^C{^1^H} NMR (C_6_D_6_, 176 MHz): δ 160.3 (N*C*N), 149.2 (Ar*C*), 148.4 (Ar*C*), 135.0 (Ar*C*), 133.6 (Ar*C*), 129.3 (Ar*C*), 128.7 (Ar*C*), 125.5 (Ar*C*), 124.1 (Ar*C*), 116.9 (N*C*CH_3_), 116.7 (N*C*CH_3_), 28.8 (*C*H(CH_3_)_2_), 28.7 (*C*H(CH_3_)_2_), 24.9 (CH(*C*H_3_)_2_), 24.9 (CH(*C*H_3_)_2_), 24.1 (CH(*C*H_3_)_2_), 24.0 (CH(*C*H_3_)_2_), 9.9 (NC*C*H_3_), 9.6 (NC*C*H_3_). *The vinylic carbon resonance was not located*. ^207^Pb{^1^H} NMR ([D_8_]toluene, 84 MHz, 0 °C): δ 5449 (s). Anal. Calcd. for C_60_H_82_N_4_Pb: C 67.57, H 7.75, N 5.25. Found: C 67.31, H 7.87, N 5.14. M.p. 85 °C (decomp.). UV‐Vis: λ_max_=582 nm (ϵ=16000 M^−1^cm^−1^). *Compound* **5** *is thermally unstable in both solution and in the solid state at room temperature*.

**Synthesis of** ^**Me**^ **IPr⋅SiBr_2_ (6)**: To a vial containing solution of ^Me^IPr (0.147 g, 0.353 mmol) in 5 mL of THF was added a solution of [K(THF)_2_][Si(SiMe_3_)_3_] (0.152 g, 0.353 mmol) in 3 mL of THF followed by the rapid addition of SiBr_4_ (44.0 μL, 0.353 mmol). Upon the addition of SiBr_4_, the formation of a white precipitate under an orange solution was observed. The reaction mixture was stirred for 1 hour and filtered through diatomaceous earth to afford an orange filtrate. The volatiles were removed from the filtrate *in vacuo* and the resultant solid washed with 2×2 mL of cold (−30 °C) hexanes to give **6** as an orange powder (0.131 g, 61 %). ^1^H and ^13^C{^1^H} NMR spectral assignments match those reported in the literature.^[18]^

**Synthesis of (^Me^IPrCH)_2_Si: (7)**: A solution of [(^Me^IPrCH)Li]_2_ (0.204 g, 0.234 mmol) in 4 mL of toluene was added to a vial containing a slurry of ^Me^IPr⋅SiBr_2_ (0.142 g, 0.235 mmol) in 1 mL of toluene. Upon addition, the reaction mixture turned a dark yellow/brown color. After stirring for 15 minutes, the volatiles were removed *in vacuo* and the residue was extracted with 5 mL of hexanes and filtered. The dark yellow/brown filtrate was concentrated to a volume of 2 mL and placed in a −30 °C freezer for 16 hours. A few crystals were removed from the bulk sample for X‐ray crystallographic analysis. The mother liquor was decanted from the bulk crystals and the crystals dried *in vacuo* affording (^Me^IPrCH)_2_Si: (**7**) as a dark yellow crystalline solid (0.057 g, 27 %). ^1^H NMR (C_6_D_6_, 700 MHz): δ 7.30 (t, 2H, ^3^ *J*_HH_=8.0 Hz, *p*‐Ar*H*), 7.25 (t, 2H, ^3^ *J*_HH_=8.0 Hz, *p*‐Ar*H*), 7.13‐7.09 (m, 8H, *m*‐Ar*H*), 4.25 (s, 2H, C*H*Si), 3.11 (sept, 4H, ^3^ *J*_HH_=7.0 Hz, C*H*(CH_3_)_2_), 2.96 (sept, 4H, ^3^ *J*_HH_=7.0 Hz, C*H*(CH_3_)_2_), 1.62 (s, 6H, NC‐C*H*_3_), 1.54 (s, 6H, NC‐C*H*_3_), 1.32 (d, 12H, ^3^ *J*_HH_=7.0 Hz, CH(C*H*_3_)_2_), 1.23 (d, 12H, ^3^ *J*_HH_=7.0 Hz, CH(C*H*_3_)_2_), 1.17 (d, 12H, ^3^ *J*_HH_=7.0 Hz, CH(C*H*_3_)_2_), 1.14 (d, 12H, ^3^ *J*_HH_=7.0 Hz, CH(C*H*_3_)_2_). ^13^C{^1^H} NMR (C_6_D_6_, 176 MHz): δ 158.1 (N*C*N), 148.8 (Ar*C*), 147.7 (Ar*C*), 134.9 (Ar*C*), 132.9 (Ar*C*), 129.2 (Ar*C*), 128.9 (Ar*C*), 124.8 (Ar*C*), 123.9 (Ar*C*), 118.3 (N*C*CH_3_), 116.9 (N*C*CH_3_), 100.0 (C=*C*H), 28.9 (*C*H(CH_3_)_2_), 28.8 (*C*H(CH_3_)_2_), 24.5 (CH(*C*H_3_)_2_), 23.8 (CH(*C*H_3_)_2_), 9.8 (NC*C*H_3_), 9.3 (NC*C*H_3_). ^29^Si{^1^H} NMR (C_6_D_6_, 79.4 MHz): δ 271.9 (s). Anal. Calcd. for C_60_H_82_N_4_Si: C 81.21, H 9.31, N 6.31. Found: C 80.20, H 9.77, N 5.69. M.p. 155–157 °C (decomp.). UV‐Vis: λ_max_=425 nm (ϵ=6500 M^−1^cm^−1^), 484 nm (ϵ=14700 M^−1^cm^−1^).

**Synthesis of (^Me^IPrCH)_2_Si(dba) (8)**: To a solution of (^Me^IPrCH)_2_Si: (0.035 g, 0.039 mmol) in 1 mL of toluene was added a solution of dibenzylideneacetone (dba) (0.009 g, 0.04 mmol) in 1 mL of toluene. After 5 minutes of stirring the reaction mixture turned light yellow. After an additional 25 minutes of stirring the volatiles were removed *in vacuo* to afford a yellow residue. The product was extracted with 2 mL of pentane and filtered. The light‐yellow filtrate was concentrated to a volume of *ca*. 1 mL and placed in a −30 °C freezer for 16 hours to afford (^Me^IPrCH)_2_Si(dba) (**8**) as a yellow crystalline solid (0.012 g, 27 %). Crystals suitable for X‐ray diffraction analysis were obtained by dissolving **8** in a minimal amount of pentane and storing the solution at −30 °C for one week. ^1^H NMR (C_6_D_6_, 700 MHz): δ 7.56 (d, 2H, ^3^ *J*_HH_=7.4 Hz, *o*‐Ph), 7.42 (d, 2H, ^3^ *J*_HH_=7.7 Hz, *o*‐Ph*H*), 7.34 (m, 3H, *m*‐Ph*H* and *p*‐Ph*H*), 7.28 (m, 3H, *m*‐Ph*H* and *p*‐Ph*H*), 7.21 (dd, 1H, ^3^ *J*_HH_=7.7, 1.2 Hz, *p*‐Dipp*H*), 7.17 (m, 2H, *m*‐Dipp*H*), 7.14 (m, 2H, *m*‐Dipp*H*), 7.12‐7.10 (m, 4H, *m*‐Dipp*H*), 7.01 (dd, 1H, ^3^ *J*_HH_=7.7, 1.2 Hz, *p*‐Dipp*H*), 6.97 (dd, 1H, ^3^ *J*_HH_=7.7, 1.2 Hz, *p*‐Dipp*H*), 6.93 (dd, 1H, ^3^ *J*_HH_=7.6, 1.1 Hz, *p*‐Dipp*H*), 6.12 (d, 1H, ^3^ *J*_HH_=15.8 Hz, Ph(H)C=C(*H*)CO), 5.85 (d, 1H, ^3^ *J*_HH_=15.8 Hz, Ph(*H*)C=C(H)CO), 4.63 (d, 1H, ^3^ *J*_HH_=3.1 Hz, OC=C(*H*)), 3.19 (m, 3H, C*H*(CH_3_)_2_), 3.13 (m, 2H, C*H*(CH_3_)_2_), 3.02 (sept, 1H, ^3^ *J*_HH_=7.0 Hz, C*H*(CH_3_)_2_), 2.93 (sept, 1H, ^3^ *J*_HH_=7.0 Hz, C*H*(CH_3_)_2_), 2.84 (sept, 1H, ^3^ *J*_HH_=7.0 Hz, C*H*(CH_3_)_2_), 2.56 (s, unresolved doublet), 1H, SiC*H*(Ph)), 2.27 (s, 1H, SiC*H*=IPr^Me^), 1.67 (s, 1H, SiC*H*=IPr^Me^), 1.61 (d, 3H, ^3^ *J*_HH_=7.0 Hz, CH(C*H*_3_)_2_), 1.45 (d, 3H, ^3^ *J*_HH_=7.0 Hz, CH(C*H*_3_)_2_), 1.43 (d, 3H, ^3^ *J*_HH_=7.0 Hz, CH(C*H*_3_)_2_), 1.41 (s, 3H, C*H*_3_), 1.36 (s, 3H, C*H*_3_), 1.35 (s, 3H, C*H*_3_), 1.25 (d, 3H, ^3^ *J*_HH_=7.0 Hz, CH(C*H*_3_)_2_), 1.22 (d, 3H, ^3^ *J*_HH_=7.0 Hz, CH(C*H*_3_)_2_), 1.21 (d, 3H, ^3^ *J*_HH_=7.0 Hz, CH(C*H*_3_)_2_), 1.20 (s, 3H, C*H*_3_), 1.19 (d, 3H, ^3^ *J*_HH_=7.0 Hz, CH(C*H*_3_)_2_), 1.18 (d, 3H, ^3^ *J*_HH_=7.0 Hz, CH(C*H*_3_)_2_), 1.09 (d, 3H, ^3^ *J*_HH_=7.0 Hz, CH(C*H*_3_)_2_), 1.06 (d, 3H, ^3^ *J*_HH_=7.0 Hz, CH(C*H*_3_)_2_), 1.02 (d, 6H, ^3^ *J*_HH_=7.0 Hz, CH(C*H*_3_)_2_), 1.01 (d, 3H, ^3^ *J*_HH_=7.0 Hz, CH(C*H*_3_)_2_), 0.97 (d, 3H, ^3^ *J*_HH_=7.0 Hz, CH(C*H*_3_)_2_), 0.88 (d, 3H, ^3^ *J*_HH_=7.0 Hz, CH(C*H*_3_)_2_), 0.80 (d, 3H, ^3^ *J*_HH_=7.0 Hz, CH(C*H*_3_)_2_). ^13^C{^1^H} NMR (C_6_D_6_, 176 MHz): δ 154.4 (N*C*N in ^Me^IPr), 154.4 (N*C*N in ^Me^IPr), 154.2 (*C*O), 149.1 (*C*CH(CH_3_)_2_), 149.0 (*C*CH(CH_3_)_2_), 148.8 (*C*CH(CH_3_)_2_), 148.6 (*C*CH(CH_3_)_2_), 148.4 (*C*CH(CH_3_)_2_), 147.8 (*C*CH(CH_3_)_2_), 147.7 (*C*CH(CH_3_)_2_), 147.3 (*C*CH(CH_3_)_2_), 145.7 (*ipso‐*C_Ph_), 139.2 (*ipso‐C*_Ph_), 135.4 (*ipso‐C*_Dipp_), 135.1 (*ipso‐C*_Dipp_), 133.6 (*ipso‐C*_Dipp_), 133.4 (*ipso‐C*_Dipp_), 130.2 (C_Ph_), 129.3 (C_Ph_), 129.2 (C_Ph_), 129.1 (C_Ph_), 129.0 (C_Ph_), 128.9 (C_Ph_), 128.5 (C_Ph_), 128.2 (C_Ph_), 127.6 (PhC(H)=*C*(H)CO), 127.5 (C_Dipp_), 126.6 (C_Dipp_), 126.5 (C_Ph_), 126.1 (C_Ph_), 125.8 (C_Dipp_), 124.8 (C_Dipp_), 124.7 (C_Dipp_), 124.6 (C_Dipp_), 124.5 (C_Dipp_), 124.4 (C_Dipp_), 124.3 (C_Dipp_), 124.2(C_Dipp_), 124.1 (Ph*C*(H)=C(H)CO), 123.9 (C_Dipp_), 123.7 (C_Dipp_), 118.2 (H_3_C*C*=CCH_3_), 118.0 (H_3_C*C*=CCH_3_), 117.2 (H_3_C*C*=CCH_3_), 117.1 (H_3_C*C*=CCH_3_), 111.2 (OC=*C*(H)), 53.9 (Si*C*H=IPr^Me^), 49.2 (Si*C*H=IPr^Me^), 38.9 (Si*C*H(Ph)), 29.1 (*C*H(CH_3_)_2_), 28.8 (*C*H(CH_3_)_2_), 28.8 (*C*H(CH_3_)_2_), 28.7 (*C*H(CH_3_)_2_), 28.7 (*C*H(CH_3_)_2_), 28.6 (*C*H(CH_3_)_2_), 28.4 (*C*H(CH_3_)_2_), 28.3 (*C*H(CH_3_)_2_), 25.9 (CH(*C*H_3_)_2_), 25.7 (CH(*C*H_3_)_2_), 25.5 (CH(*C*H_3_)_2_), 24.8 (CH(*C*H_3_)_2_), 24.7 (CH(*C*H_3_)_2_), 24.5 (CH(*C*H_3_)_2_), 24.5 (CH(*C*H_3_)_2_), 24.4 (CH(*C*H_3_)_2_), 24.2 (CH(*C*H_3_)_2_), 24.0 (CH(*C*H_3_)_2_), 24.0 (CH(*C*H_3_)_2_), 23.9 (CH(*C*H_3_)_2_), 23.8 (CH(*C*H_3_)_2_), 23.5 (CH(*C*H_3_)_2_), 23.3 (CH(*C*H_3_)_2_), 22.7 (CH(*C*H_3_)_2_), 10.4 (NC*C*H_3_), 10.2 (NC*C*H_3_), 10.1 (2×NC*C*H_3_). Anal. Calcd. for C_77_H_96_N_4_OSi: C 82.45, H 8.63, N 4.99; Found: C 81.33, H 8.19, N 4.05. M.p. 229–232 °C.

**Synthesis of [ImMe_2_** *i* **Pr_2_‐CH_3_]I (9)**: 2,4,5‐Trimethylimidazole (2.595 g, 23.56 mmol) and K_2_CO_3_ (7.243 g, 52.41 mmol) were dissolved in 30 mL of acetonitrile and the mixture was heat to reflux for 2 hours. The reaction mixture was allowed to cool to room temperature, then *i*PrI (6.0 mL, 60 mmol) was added. The reaction mixture was then heat to reflux again for 48 hours, cooled to room temperature and the volatiles removed under vacuum. The resulting product was then extracted with 200 mL of CH_2_Cl_2_ and the extract filtered; the remaining solid was washed with another 50 mL of CH_2_Cl_2_ and the extract was filtered. The solvent was then removed from the combined CH_2_Cl_2_ extracts under vacuum to give a black oil. This oil was triturated with 500 mL of Et_2_O to yield [ImMe_2_ *i*Pr_2_‐CH_3_]I (**9**) as a light brown solid, which was isolated by filtration and dried (2.520 g, 33 %). ^1^H NMR (CD_3_CN, 700 MHz,): δ 4.70 (sept, 2H, ^3^ *J*_HH_=7.0 Hz, C*H*(CH_3_)_2_), 2.65 (s, 3H, ImMe_2_ *i*Pr_2_‐C*H*_3_), 2.26 (s, 6H, ‐NC(C*H*_3_)), 1.51 (d, 12H, ^3^ *J*_HH_=7.0 Hz, CH(C*H*_3_)_2_). ^13^C{^1^H} NMR (CD_3_CN, 176 MHz): δ 142.4 (N*C*N), 126.6 (N*C*(CH_3_)), 51.3 (ImMe_2_ *i*Pr_2_‐*C*H_3_), 21.3 (CH(*C*H_3_)_2_), 13.0 (*C*H(CH_3_)_2_), 10.3 (NC(*C*H_3_)). Anal. Calcd. for C_12_H_23_IN_2_: C 44.73, H 7.19, N 8.69; Found: C 44.49, H 7.04, N 8.46. M.p. 135–137 °C.

**Synthesis of ImMe_2_** *i* **Pr_2_=CH_2_ (10)**: To a solution of [ImMe_2_ *i*Pr_2_‐CH_3_]I (0.164 g, 0.510 mmol) in 4 mL of THF was added rapidly a solution of K[N(SiMe_3_)_2_] (0.102 g, 0.509 mmol) in 4 mL of THF. Upon addition of K[N(SiMe_3_)_2_] a white precipitate was observed. After stirring for an additional 2 hours the volatiles were removed under vacuum to afford a brown residue. The product was extracted with 10 mL of toluene and the extract filtered through diatomaceous earth to yield a clear orange filtrate. The solvent was removed from the filtrate under vacuum to give ImMe_2_ *i*Pr=CH_2_ (**10**) as a dark brown oil (0.076 g, 77 %). ^1^H NMR (C_6_D_6_, 400 MHz): δ 3.89 (sept, 2H, ^3^ *J*_HH_=4.0 Hz, C*H*(CH_3_)_2_), 2.98 (s, 2H, C=C*H*_2_), 1.66 (s, 6H, NC(C*H*_3_)), 1.23 (d, 12H, ^3^ *J*_HH_=4.0 Hz, CH(C*H*_3_)_2_). ^13^C{^1^H} NMR (C_6_D_6_, 176 MHz): δ 128.1 (N*C*N), 114.8 (N*C*(CH_3_)), 45.8 (CH(*C*H_3_)_2_), 43.2 (C=*C*H_2_), 19.5 (*C*H(CH_3_)_2_), 10.1 (NC(*C*H_3_)). Anal. Calcd. for C_12_H_22_N_2_: C 74.17, H 11.41, N 14.42; Found: C 73.40, H 11.38, N 14.15. *Attempts to prepare the iodide salt [ImMe_2_^i^Pr_2_‐CH_2_I]I following the established procedure used to prepare* ^*Me*^ *IPr=CH(I) were unsuccessful. A mixture of [ImMe_2_iPr_2_‐CH_2_I]I and [ImMe_2_iPr_2_‐CH_2_I]I_3_ was likely obtained and could not be separated due to similar solubilities; attempts to deprotonate this salt mixture and afford ImMe_2_iPr_2_=CH(I) were unsuccessful*.

## Conflict of interest

The authors declare no conflict of interest.

## Supporting information

As a service to our authors and readers, this journal provides supporting information supplied by the authors. Such materials are peer reviewed and may be re‐organized for online delivery, but are not copy‐edited or typeset. Technical support issues arising from supporting information (other than missing files) should be addressed to the authors.

Supplementary
