# Supplementary material for: A Stable Homoleptic Divinyl Tetrelene Series
Source: Chemistry. 2021 May 14;27(33):8572–9. doi: 10.1002/chem.202100969 (PMC8252546; doi:10.1002/chem.202100969)
Supplement: Supplementary file 1 — Supplementary [file CHEM-27-8572-s001.pdf]

Chemistry–A European Journal

Supporting Information

A Stable Homoleptic Divinyl Tetrelene Series

Matthew M. D. Roy, Samuel R. Baird, Eike Dornsiepen, Lucas A. Paul, Linkun Miao, Michael J. Ferguson, Yuqiao Zhou, Inke Siewert,* and Eric Rivard*

Contents:

NMR Spectroscopic Data
NMR Spectra for ^{Me}IPr=CH(I) (**1**) S2
NMR Spectra for [(^{Me}IPrCH)Li$]_2$ (**2**) S4
NMR Spectra for (^{Me}IPrCH$)_2$Sn: (**4**) S8
NMR Spectra for (^{Me}IPrCH$)_2$Pb: (**5**) S11
NMR Spectra for (^{Me}IPrCH$)_2$Si: (**7**) S14
NMR Spectra for (^{Me}IPrCH$)_2$Si(dba) (**8**) S17
NMR Spectra for [ImMe$_2{}^i$Pr$_2$-CH$_3$]I (**9**) S20
NMR Spectra for ImMe$_2{}^i$Pr$_2$=CH$_2$ (**10**) S22
DOSY NMR Spectra of ^{Me}IPr=CH$_2$ and [(^{Me}IPrCH)Li$]_2$ (**2**) S24

X-Ray Crystallographic Data
Table S1. Crystallographic Details for ^{Me}IPr=CH(I) (**1**) S26
Figure S25. Molecular structure of ^{Me}IPr=CH(I) (**1**) S27
Table S2. Crystallographic Details for [(^{Me}IPrCH)Li$]_2$ (**2**) S28
Table S3. Crystallographic Details for (^{Me}IPrCH$)_2$Sn: (**4**) S30
Table S4. Crystallographic Details for the C-H activated Sn species **4′** S32
Figure S26. Molecular structure of **4′** S33
Table S5. Crystallographic Details for (^{Me}IPrCH$)_2$Pb: (**5**) S34
Figure S27. Molecular structure of (^{Me}IPrCH$)_2$Pb: (**5**) S35
Table S6. Crystallographic Details for (^{Me}IPrCH$)_2$Si: (**7**) S36
Table S7. Crystallographic details for (^{Me}IPrCH$)_2$Si(dba) (**8**) S38

Cyclic Voltammetry Data
Figure S28. Scan rate dependent CV data of **7** under a N_2 atmosphere S40
Figure S29. Scan rate dependent CV data of **7** under a N_2 atmosphere S41

Computational Data and UV-Vis
Figure S30. UV-Visible spectra of divinyltetrelenes (^{Me}IPrCH$)_2$E: (E = Si–Pb) S42
Table S8. TD-DFT assigned electronic transitions of the divinyltetrelenes S43
Table S9. Selected computational data for the divinyltetrelenes S44

NMR Spectra

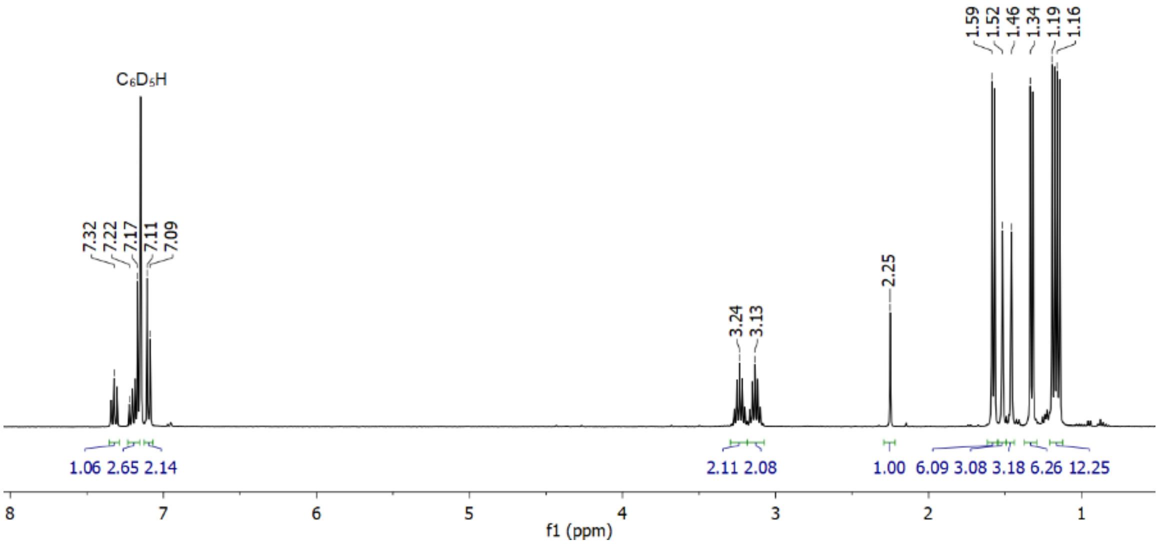

Figure S1. ^{1}H NMR spectrum of ^{Me}IPr=CH(I) (**1**) in C_6D_6.

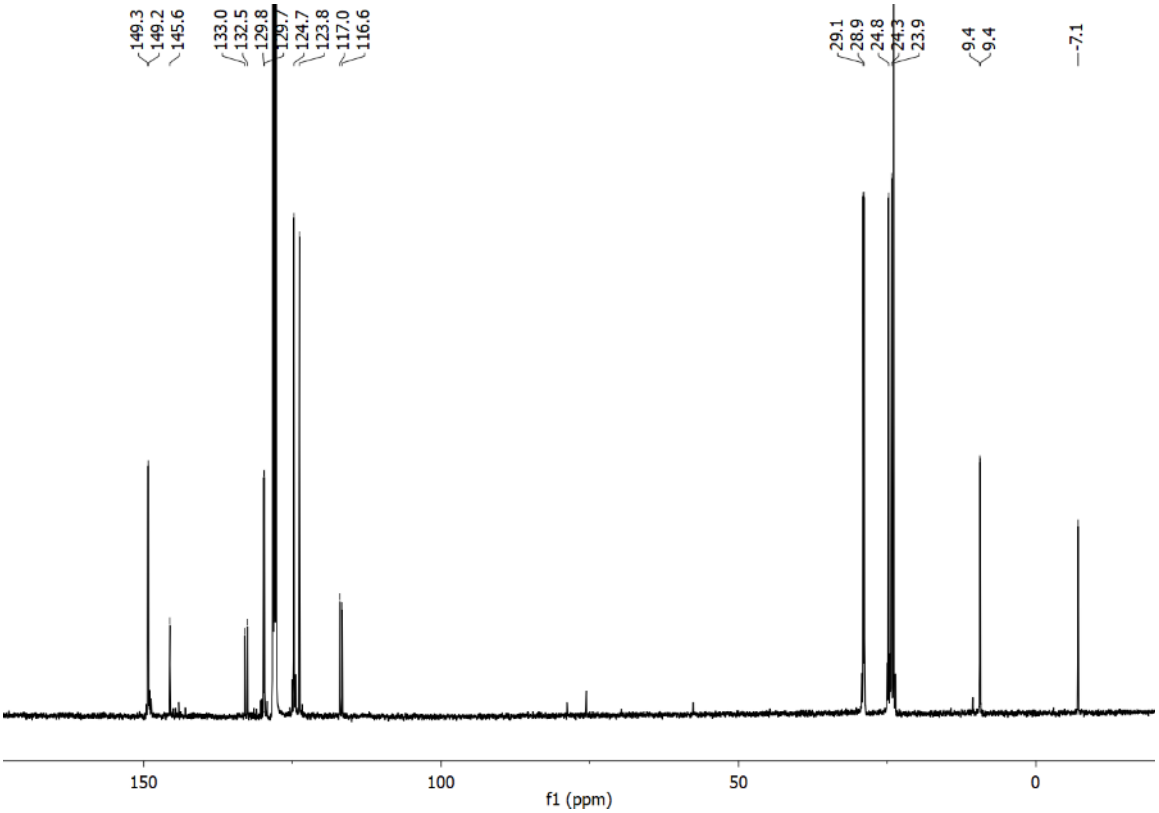

Figure S2. $^{13}C\{^1H\}$ NMR spectrum of ^{Me}IPr=CH(I) (**1**) in C_6D_6.

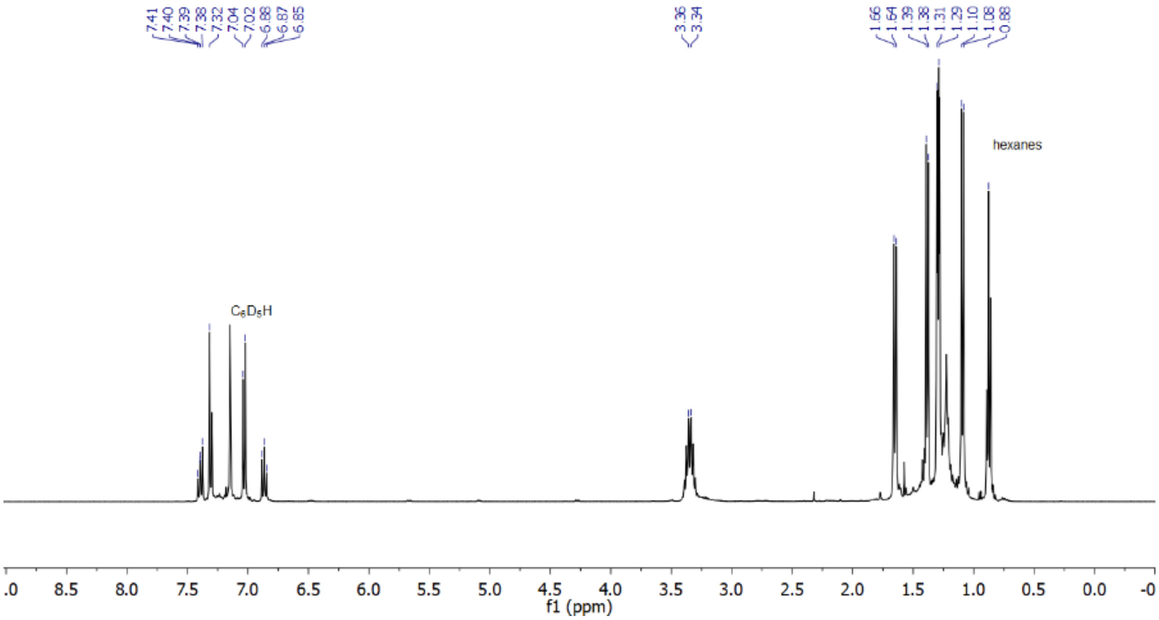

Figure S3. ¹H NMR spectrum of $[(^{Me}IPrCH)Li]_2$ (**2**) in C_6D_6.

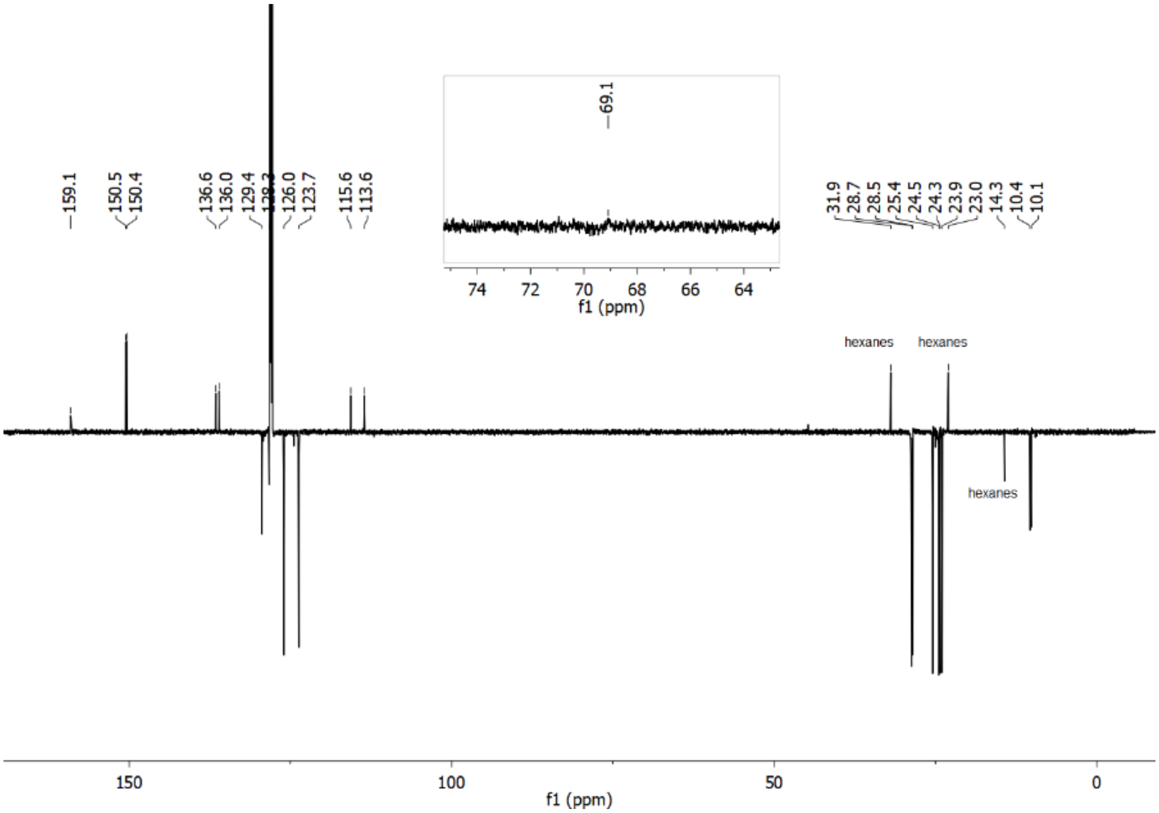

Figure S4. $^{13}C\{^{1}H\}$ DEPT NMR spectrum of $[(^{Me}IPrCH)Li]_2$ (**2**) in C_6D_6.

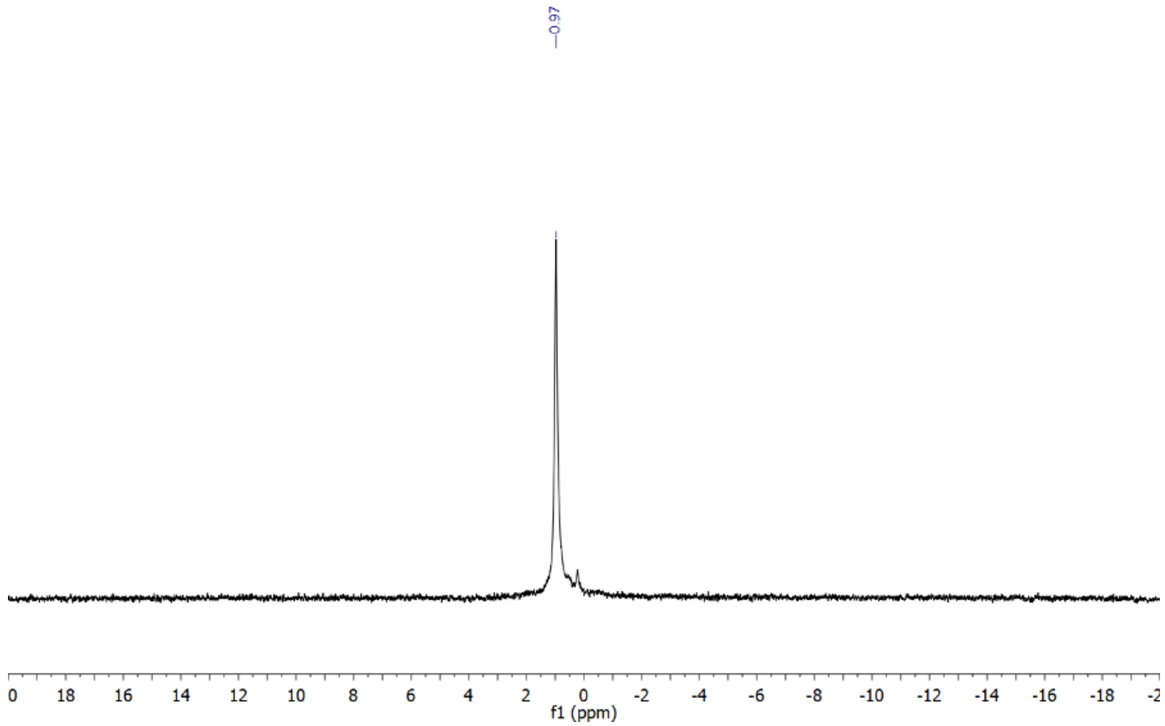

Figure S5. $^{7}Li\{^{1}H\}$NMR spectrum of $[(^{Me}IPrCH)Li]_2$ (**2**) in C_6D_6.

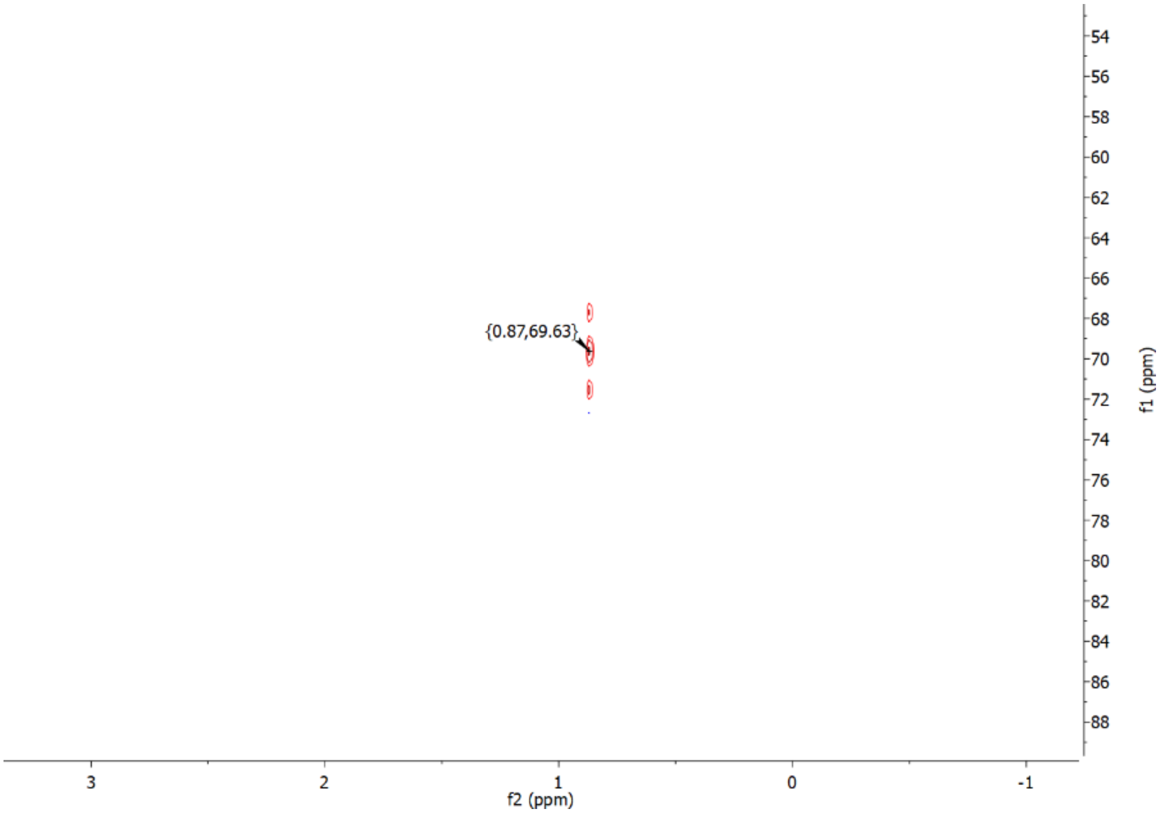

Figure S6. ^{1}H-^{13}C HSQC NMR spectrum of [(^{Me}IPrCH)Li]$_2$ (**2**) in C_6D_6.

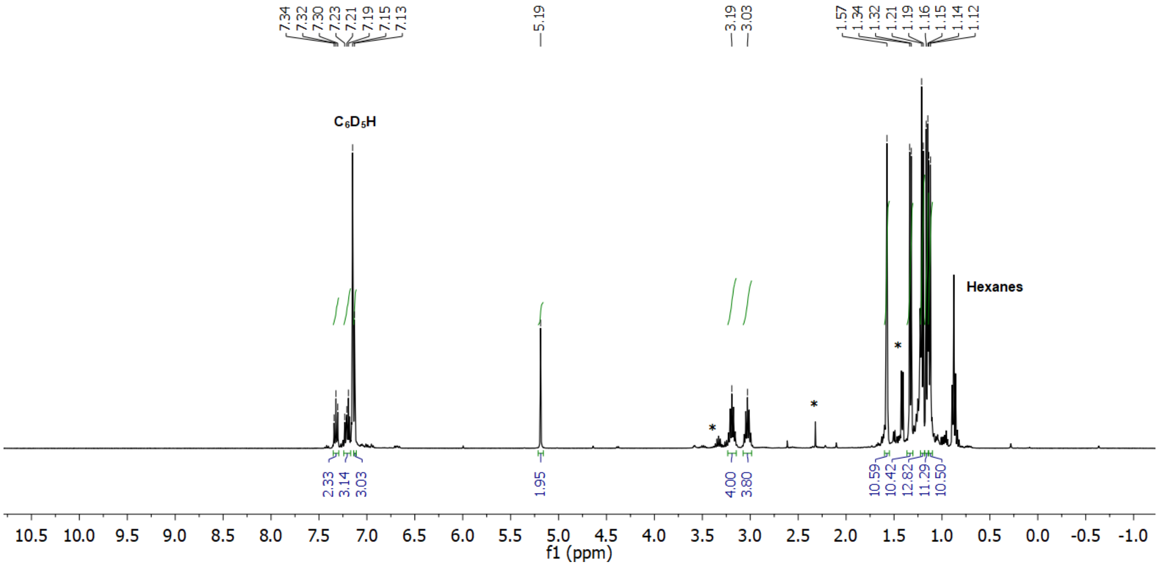

Figure S7. ^{1}H NMR spectrum (^{Me}IPrCH)$_2$Sn: (**4**) in C_6D_6. Signals marked with * belong to ^{Me}IPrCH$_2$.

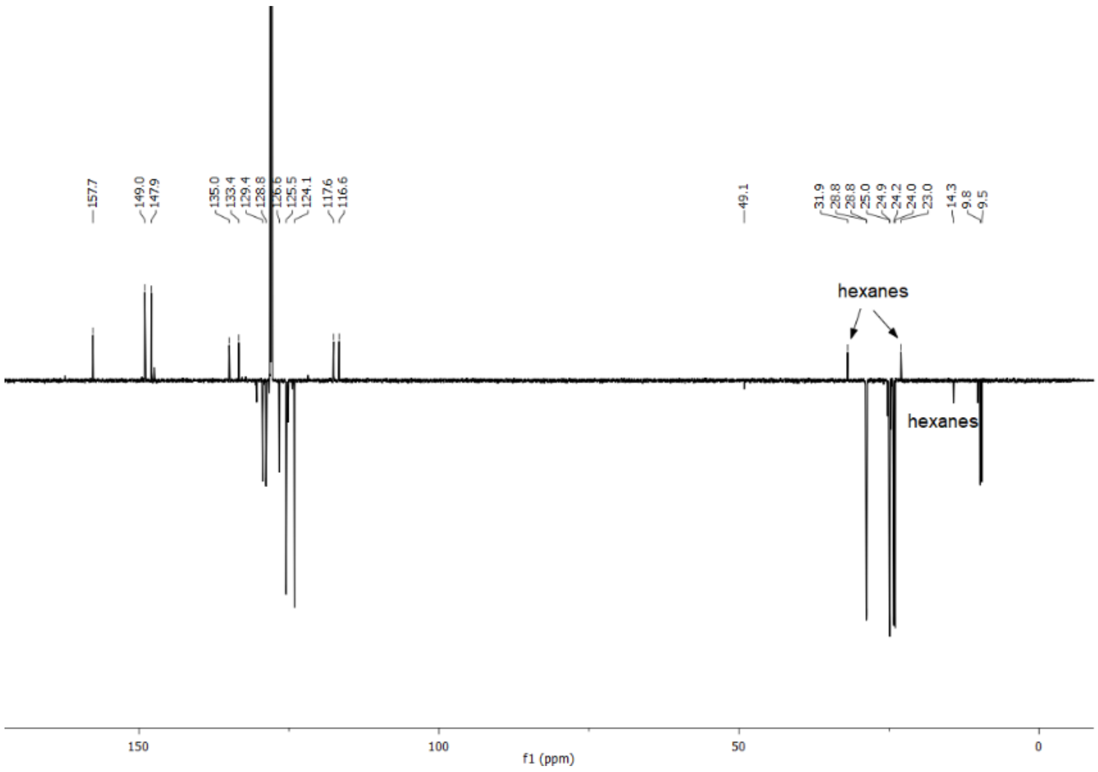

Figure S8. $^{13}C\{^1H\}$ DEPT NMR spectrum of $(^{Me}IPrCH)_2Sn$: (**4**) in C_6D_6.

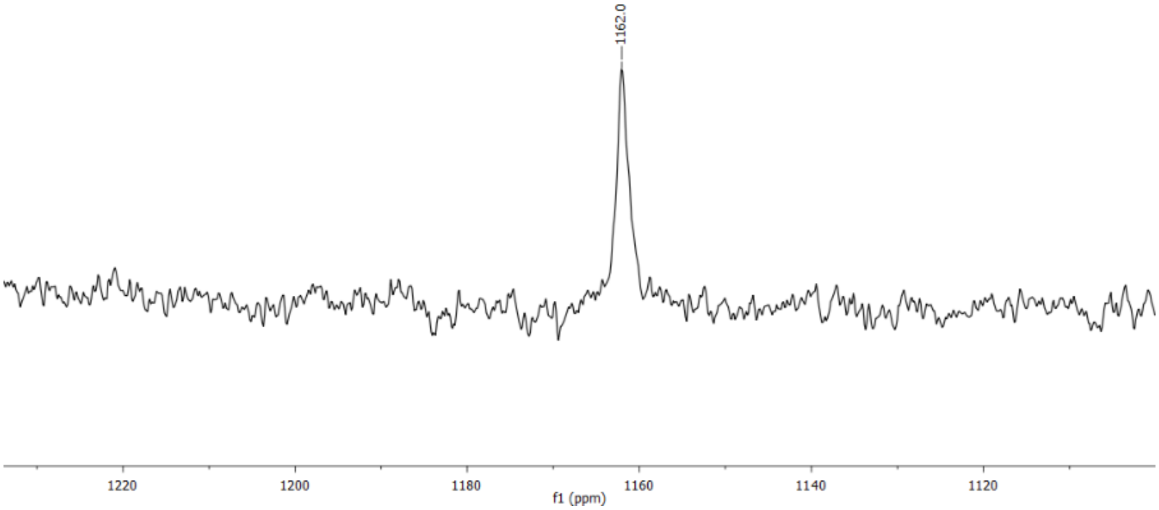

Figure S9. $^{119}Sn\{^1H\}$ NMR spectrum of $(^{Me}IPrCH)_2Sn$: (**4**) in $[D_8]$toluene.

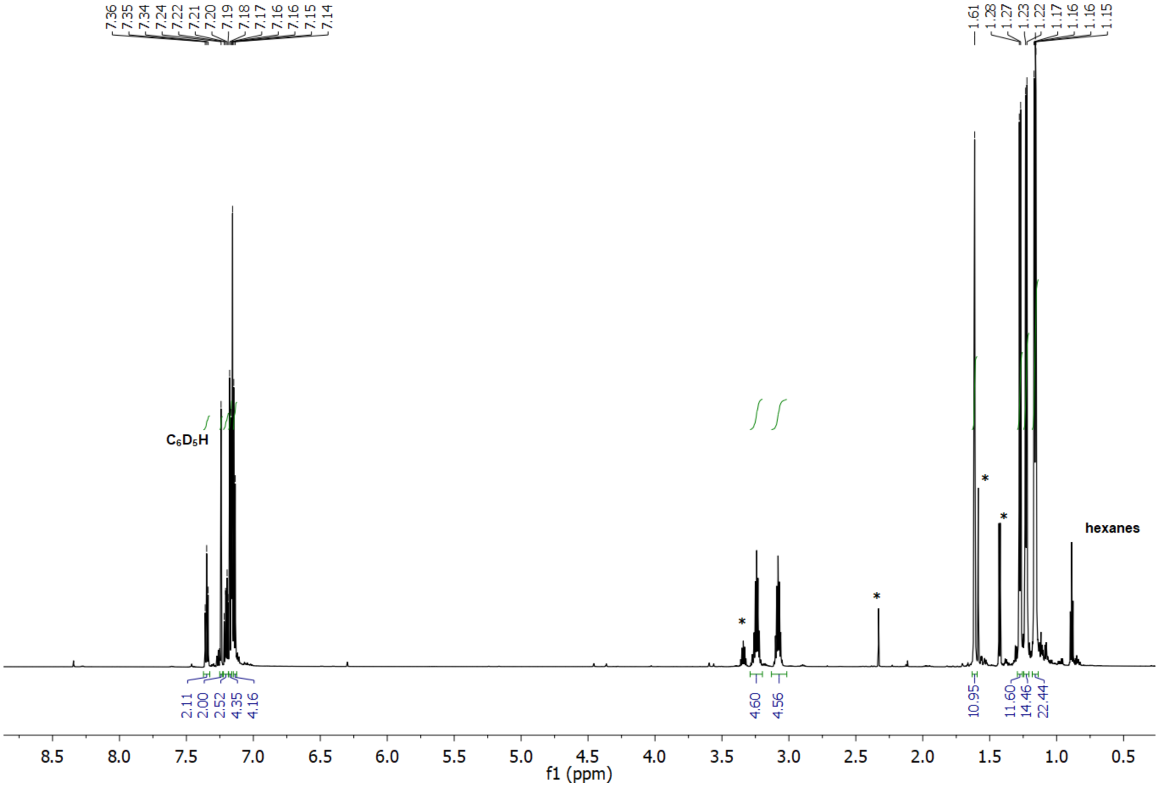

Figure S10. ^{1}H NMR spectrum of (^{Me}IPrCH)$_2$Pb: (**5**) in C_6D_6. Signals marked with * belong to ^{Me}IPrCH$_2$. Note: compound **5** is unstable in solution.

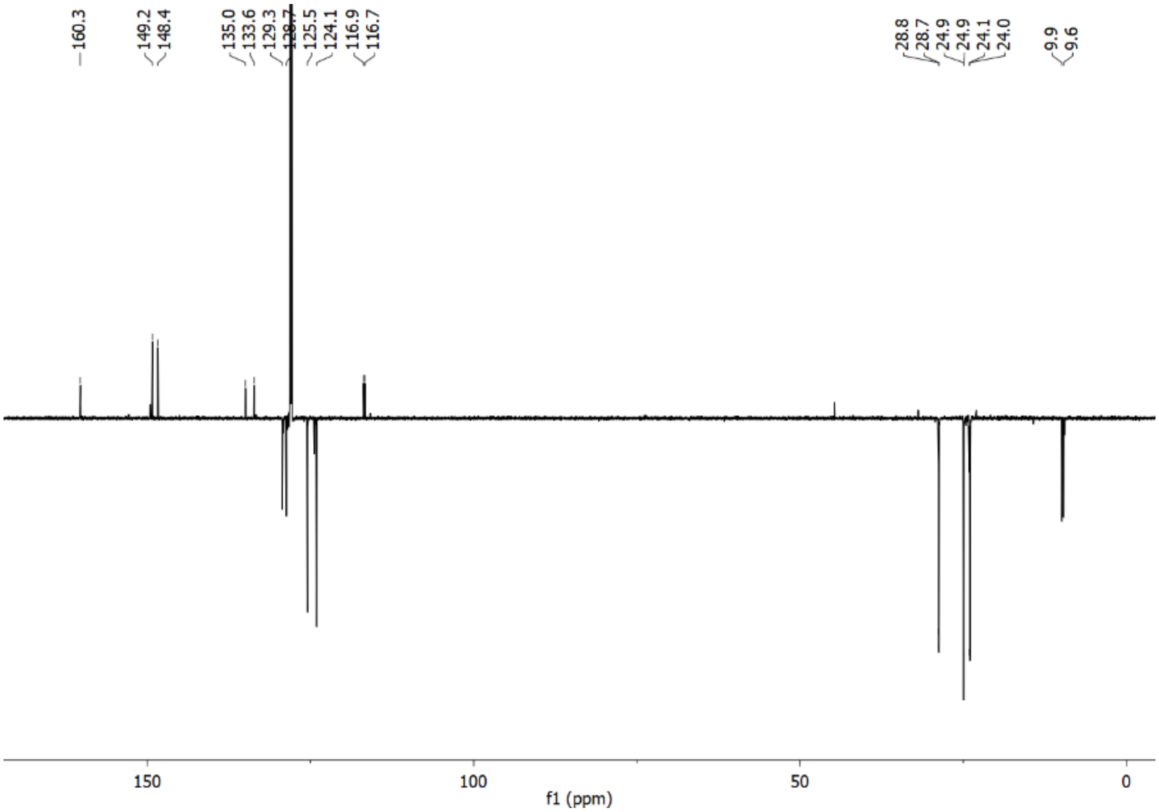

Figure S11. $^{13}C\{^{1}H\}$ DEPT NMR spectrum of $(^{Me}IPrCH)_2Pb$: (**5**) in C_6D_6.

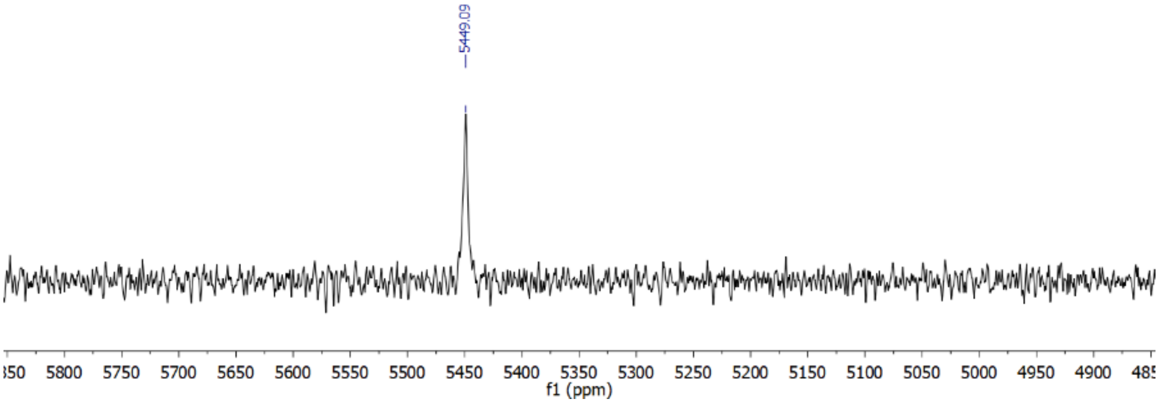

Figure S12. $^{207}Pb\{^{1}H\}$ NMR spectrum of $(^{Me}IPrCH)_2Pb$: (**5**) in [D_8]toluene.

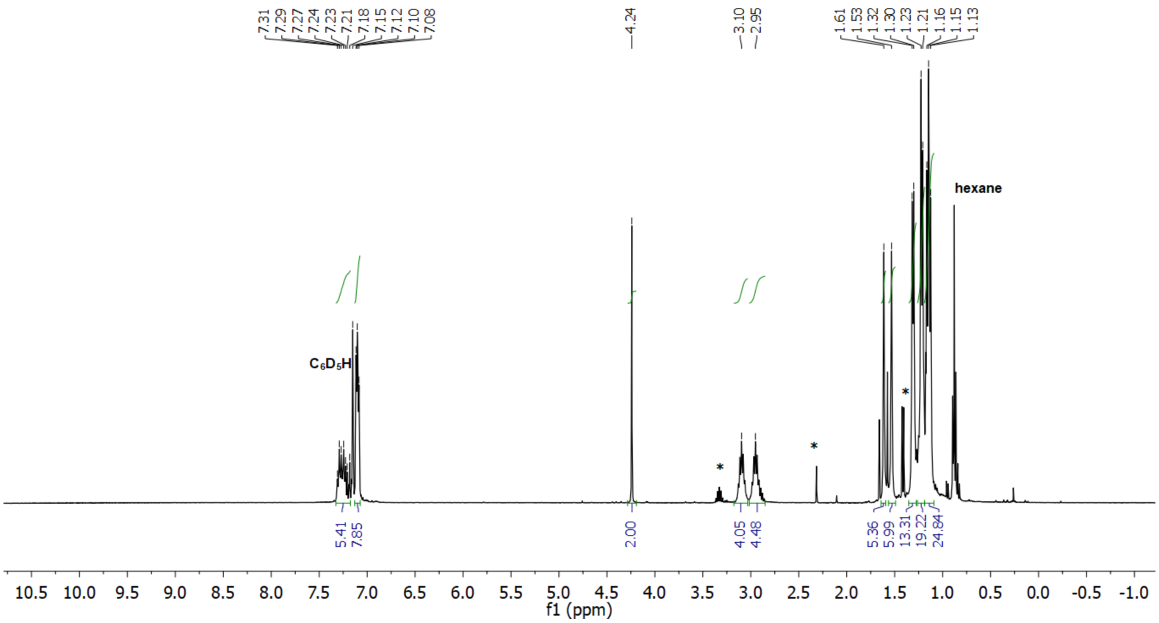

Figure S13. ^{1}H NMR spectrum of (^{Me}IPrCH)$_2$Si: (**7**) in C_6D_6. Signals marked with * belong to a minor quantity of ^{Me}IPrCH_2.

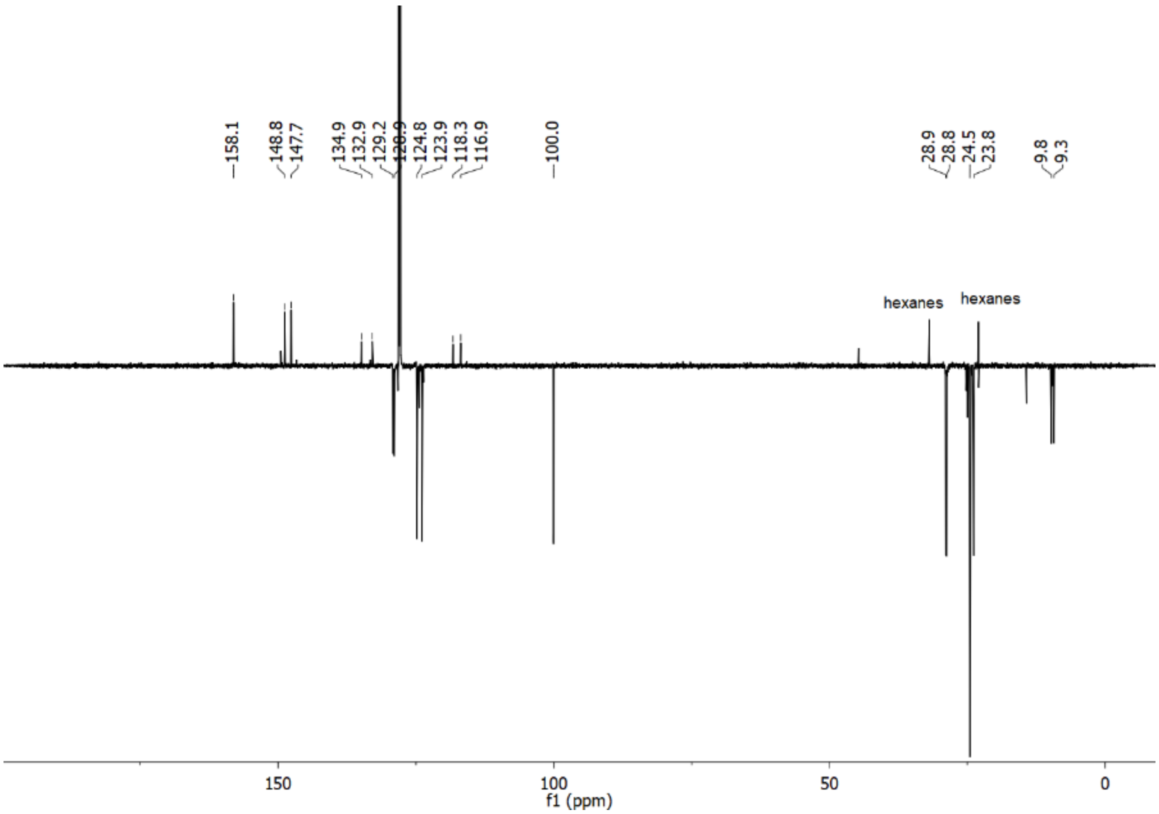

Figure S14. $^{13}C\{^1H\}$ DEPT NMR spectrum of $(^{Me}IPrCH)_2Si$: (**7**) in C_6D_6.

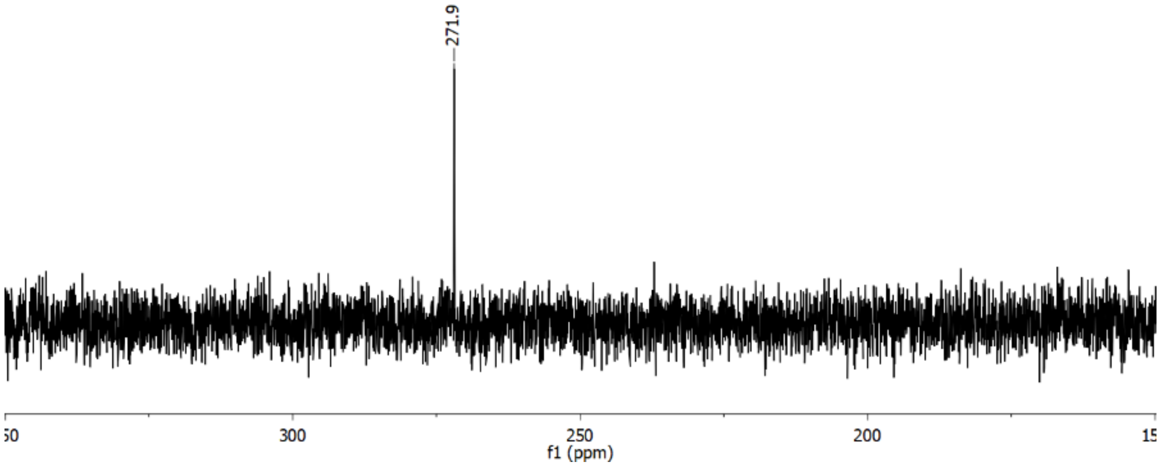

Figure S15. $^{29}Si\{^{1}H\}$ DEPT NMR spectrum of $(^{Me}IPrCH)_2Si$: (**7**) in C_6D_6.

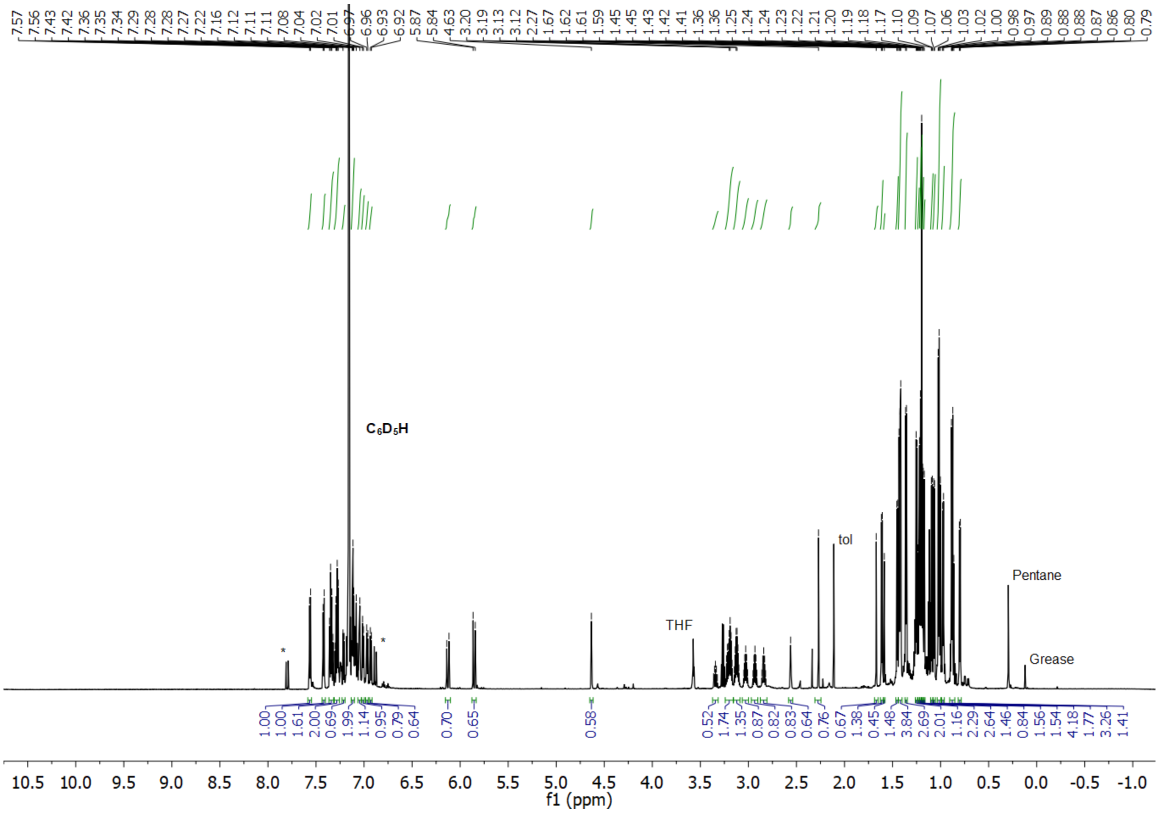

Figure S16. ^{1}H NMR spectrum of (^{Me}IPrCH)$_2$Si(dba) (**8**) in C_6D_6. Signals marked with * belong to free dba.

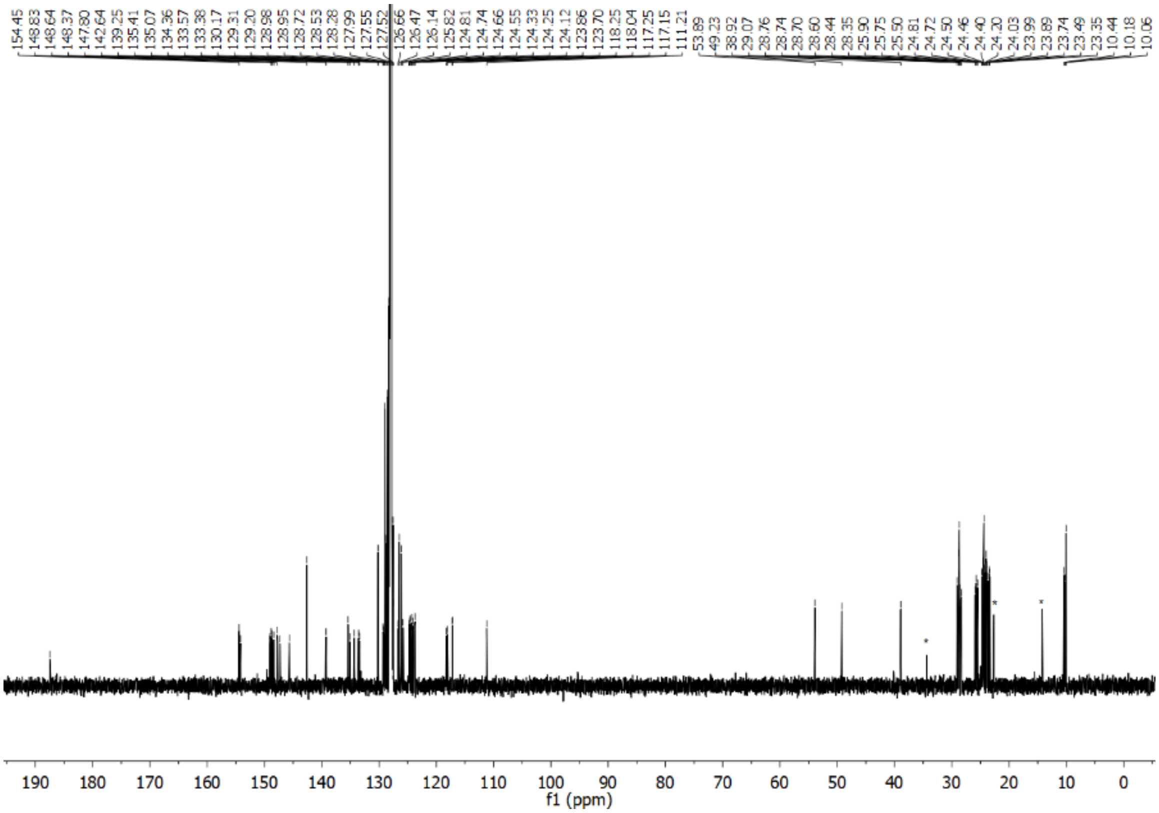

Figure S17. $^{13}C\{^1H\}$ NMR spectrum of $(^{Me}IPrCH)_2Si(dba)$ (**8**) in C_6D_6. Signals marked with * belong to pentane.

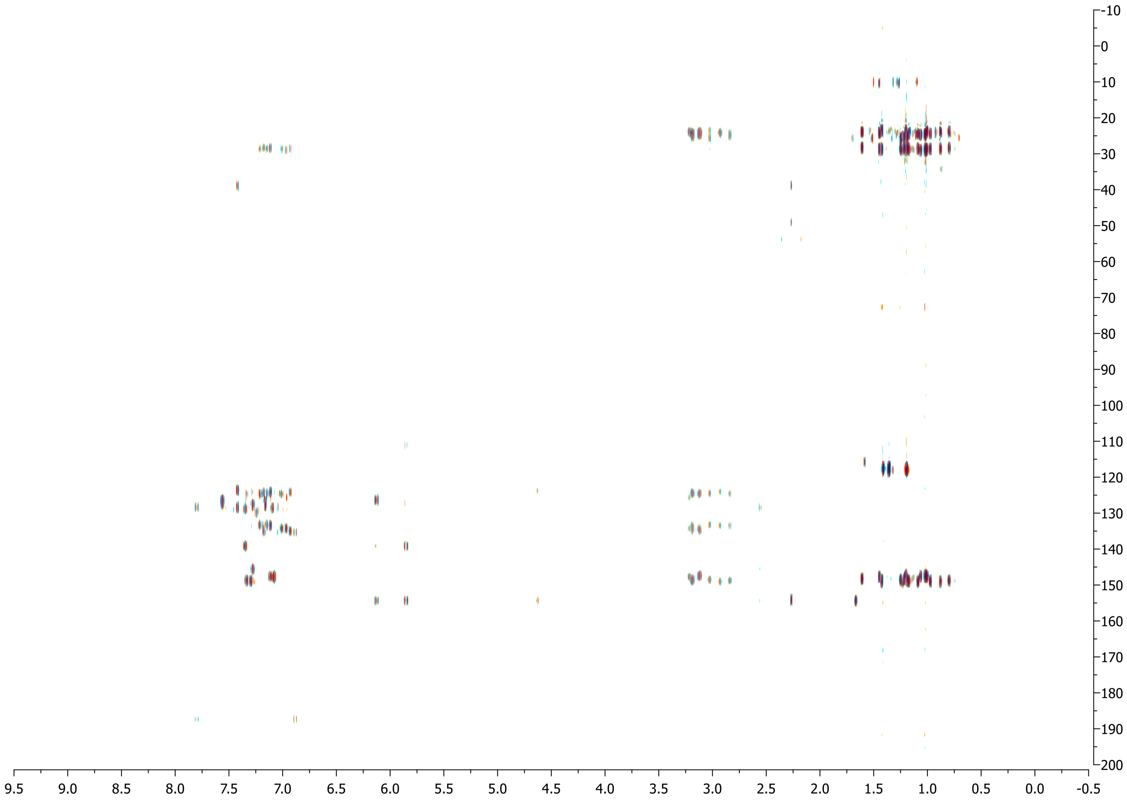

Figure S18. HMBC NMR spectrum of $(^{Me}IPrCH)_2Si(dba)$ (**8**) in C_6D_6.

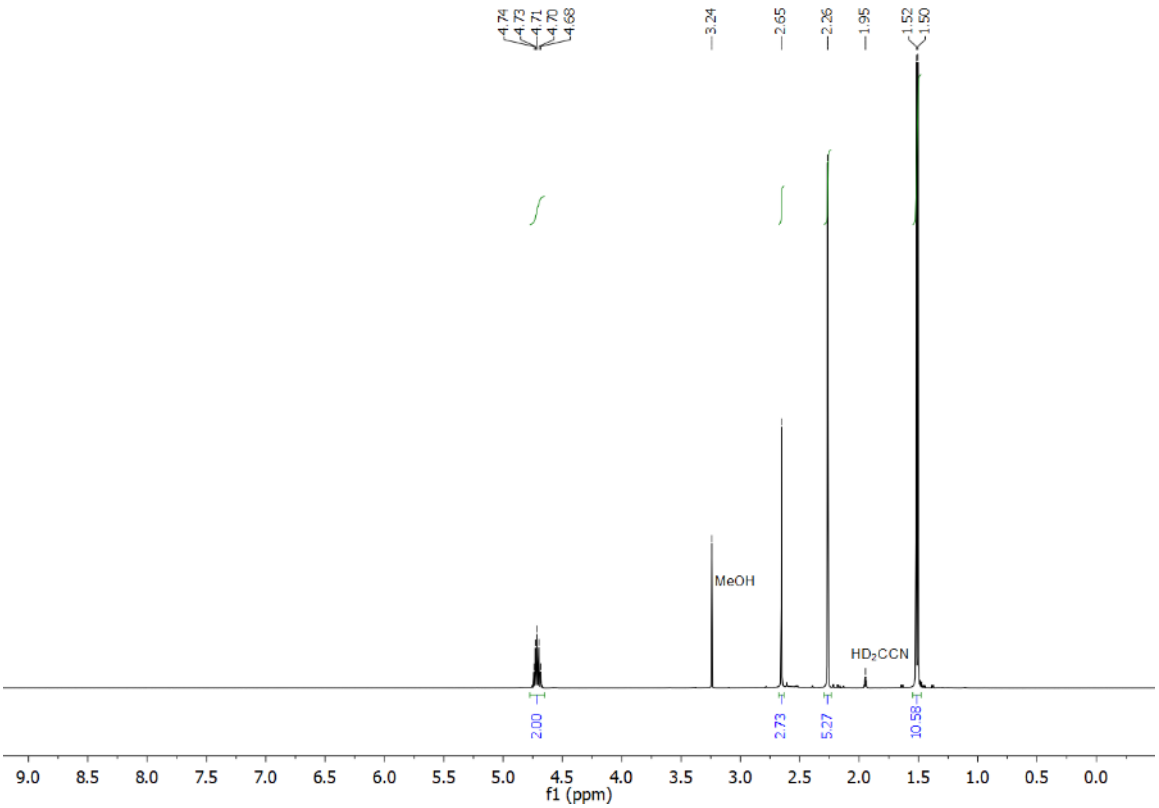

Figure S19. ^{1}H NMR spectrum of **[**ImMe$_2$^{i}Pr$_2$-CH$_3$]I (**9**) in D$_3$CCN.

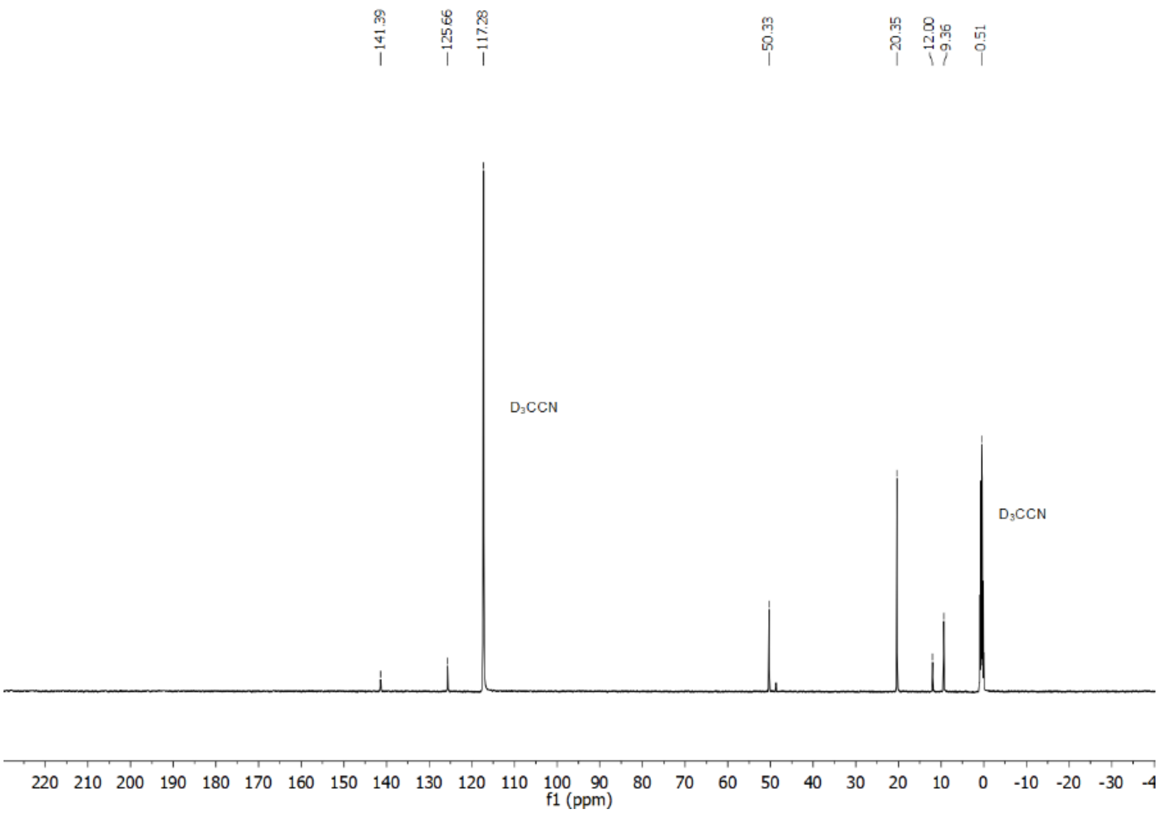

Figure S20. $^{13}C\{^1H\}$ NMR spectrum of **[**ImMe$_2$^{i}Pr$_2$-CH$_3$]I (**9**) in D_3CCN.

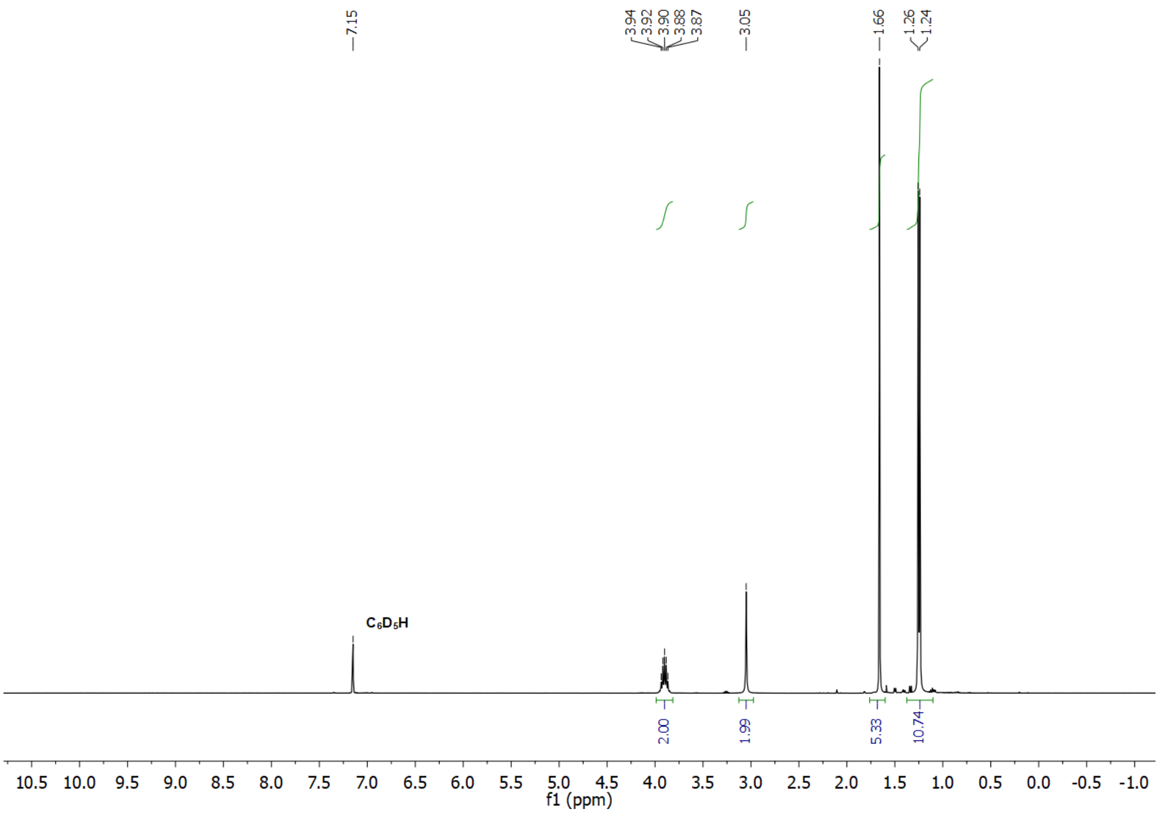

Figure S21. ^{1}H NMR spectrum of ImMe$_2{}^i$Pr$_2$=CH$_2$ (**10**) in C_6D_6.

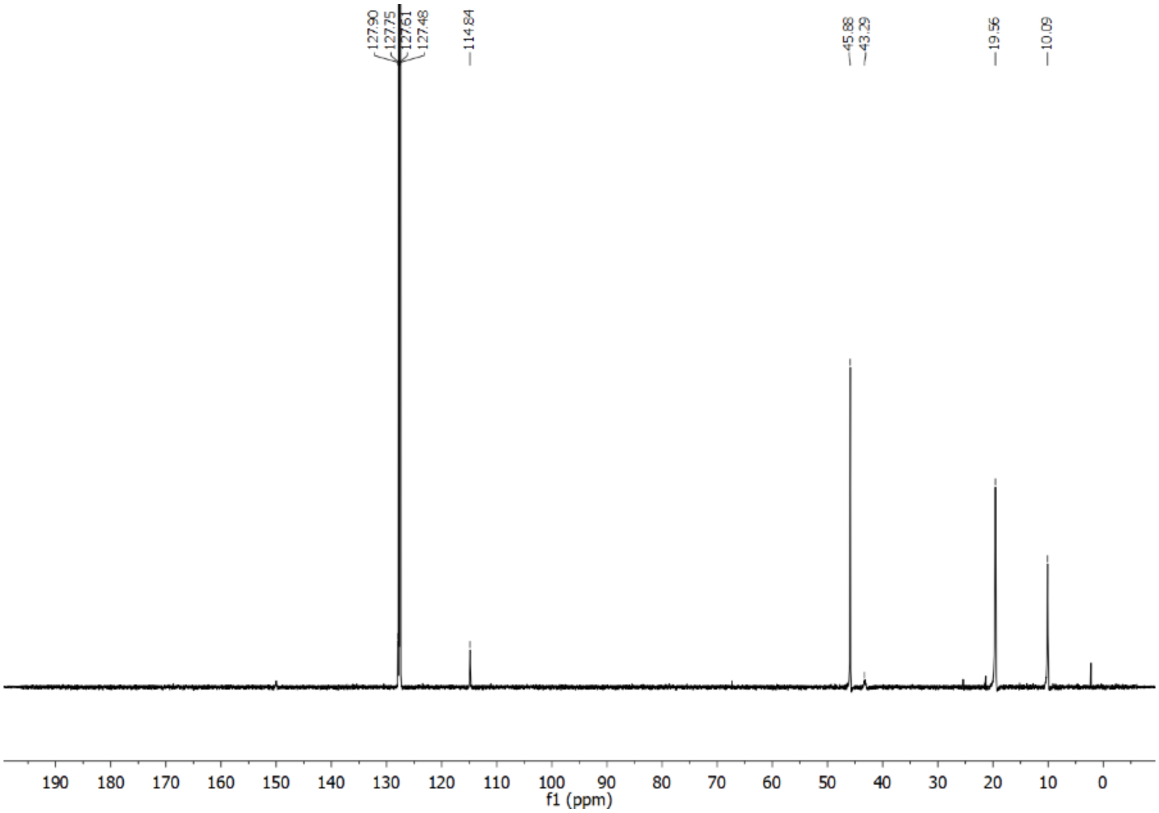

Figure S22. $^{13}C\{^1H\}$ NMR spectrum of $ImMe_2{}^iPr_2{=}CH_2$ (**10**) in C_6D_6.

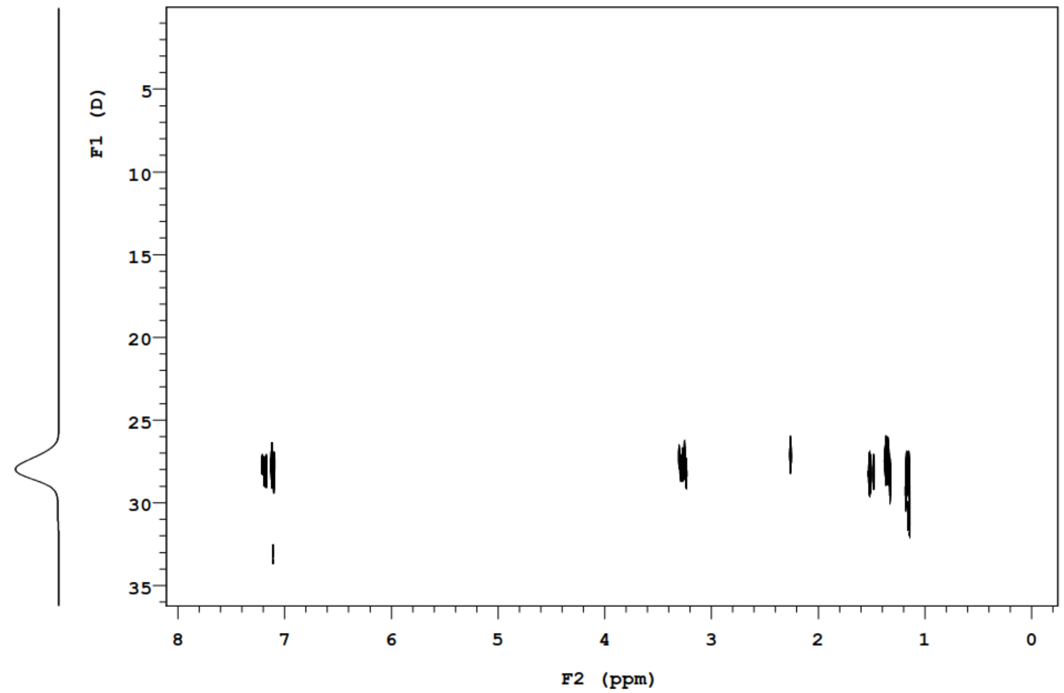

Figure S23. DOSY NMR spectrum of ^{Me}IPr=CH_2 in C_6D_6. Y-axis denotes diffusion coefficient (m^2s^{-2}).

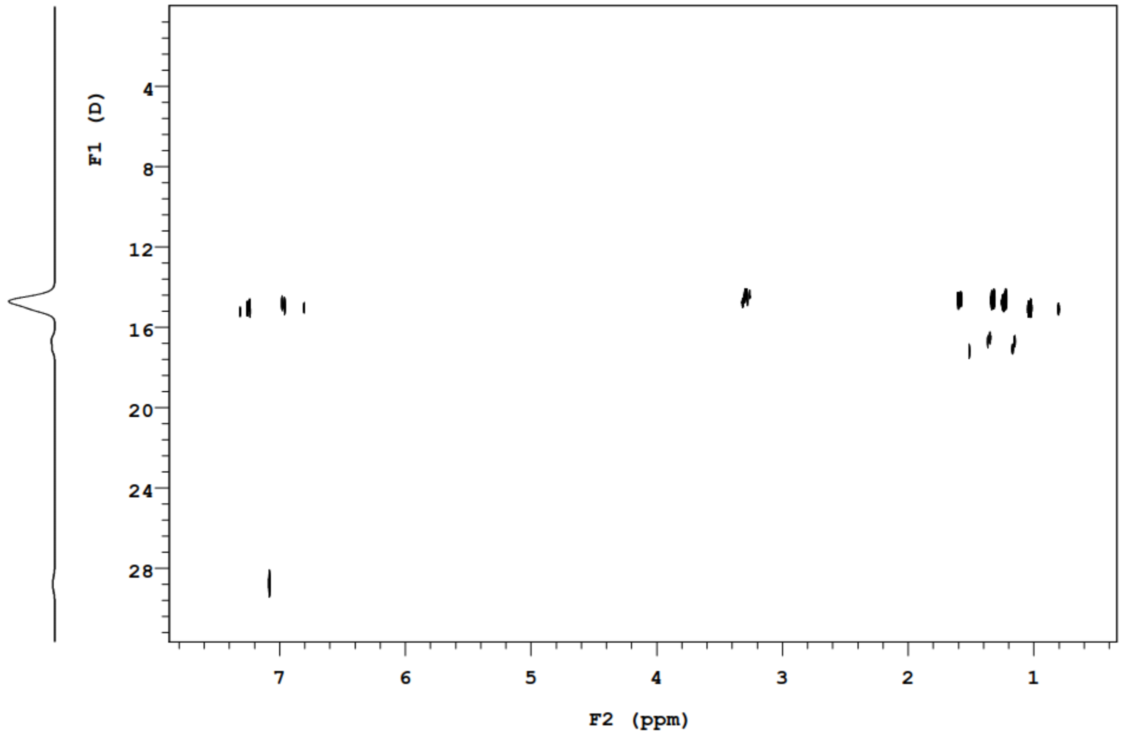

Figure S24. DOSY NMR spectrum of [(^{Me}IPrCH)Li]$_2$ in C_6D_6. Y-axis denotes diffusion coefficient ($\times 10^{-10}$ m^2s^{-2}).

$$H_r = \frac{k_b T}{6\pi\eta D}$$

H_r = hydrodynamic radius
k_b = Boltzmann constant
T = temperature of experiment = 298 K
η = viscosity of benzene at 298 K
D = experimentally determined diffusion coefficient

$$\frac{H_r\left({}^{Me}IPrCH_2\right)}{H_r\left({}^{Me}IPrCHLi\right)} = \frac{D\left({}^{Me}IPrCHLi\right)}{D\left({}^{Me}IPrCH_2\right)} = \frac{14\times10^{-10}\ m^2s^{-2}}{28\times10^{-10}\ m^2s^{-2}} = 0.5$$

The hydrodynamic radius of (^{Me}IPrCH)Li is roughly twice that of ^{Me}IPr=CH_2 in C_6D_6 solution, implying that (^{Me}IPrCH)Li is a dimer in benzene solution, in line with its solid-state structure.

X-Ray Crystallographic Data

Table S1. Crystallographic details for ^{Me}IPr=CH(I) (**1**).

A. Crystal Data	
formula	$C_{30}H_{41}IN_2$
formula weight	556.55
crystal dimensions (mm)	0.32 × 0.23 × 0.17
crystal system	triclinic
space group	$P\bar{1}$ (No. 2)]
unit cell parameters[a]	
a (Å)	9.0297(18)
b (Å)	9.5889(19)
c (Å)	18.373(4)
α (deg)	84.60(3)
β (deg)	86.53(3)
γ (deg)	64.76(3)
V (Å^3)	1432.2(6)
Z	2
ρ_{calcd} (g cm^{-3})	1.291
μ (mm^{-1})	8.905
B. Data Collection and Refinement Conditions	
diffractometer	Bruker D8/APEX II CCD[b]
radiation (λ [Å])	Cu Kα (1.54178) (microfocus source)
temperature (°C)	–100
scan type	ω and ϕ scans (1.0°) (5-5-10 s exposures)[c]
data collection 2θ limit (deg)	149.66
total data collected	5579 ($-11 \le h \le 11$, $-11 \le k \le 11$, $0 \le l \le 22$)
independent reflections	5579 (R_{int} = 0.0537)
number of observed reflections (NO)	5406 [$F_o^2 \ge 2\sigma(F_o^2)$]
structure solution method	intrinsic phasing (*SHELXT-2014*[d])
refinement method	full-matrix least-squares on F^2 (*SHELXL–2017*[e])
absorption correction method	Gaussian integration (face-indexed)
range of transmission factors	0.1340–0.0329
data/restraints/parameters	5579 / 0 / 309
goodness-of-fit (S)[f] [all data]	1.039
final R indices[g]	
R_1 [$F_o^2 \ge 2\sigma(F_o^2)$]	0.0420
wR_2 [all data]	0.1168
largest difference peak and hole	2.764 and –0.705 e Å^{-3}

^{a}Obtained from least-squares refinement of 9883 reflections with 4.82° < 2θ < 148.68°.

^{b}Programs for diffractometer operation, data collection, data reduction and absorption correction were those supplied by Bruker. The crystal used for data collection was found to display non-merohedral twinning. Both components of the twin were indexed with the program *CELL_NOW* (Bruker AXS Inc., Madison, WI, 2004). The second twin component can be related to the first component by 179.9° rotation about the [0.499 1.000 0.000] axis in real space and about the [0.144 1 -0.053] axis in reciprocal space. Integrated intensities for the reflections from the two components were written into a *SHELXL-2014* HKLF 5 reflection file with the data integration program *SAINT* (version 8.38A), using all reflection data (exactly overlapped, partially overlapped and non-overlapped). The refined value of the twin fraction (*SHELXL-2014* BASF parameter) was 0.18486.

^{c}Data were collected with the detector set at three different positions. Low-angle (detector 2θ = –33°) data frames were collected using a scan time of 5 s, medium-angle (detector 2θ = 75°) frames using a scan time of 5 s, and high-angle (detector 2θ = 117º) frames using a scan time of 10 s.

^{d}Sheldrick, G. M. *Acta Crystallogr.* 2015, **A71**, 3–8. (*SHELXT-2014*)

^{e}Sheldrick, G. M. *Acta Crystallogr.* 2015, **C71**, 3–8. (*SHELXL-2017*)

$^f S = [\Sigma w(F_o^2 - F_c^2)^2/(n - p)]^{1/2}$ (n = number of data; p = number of parameters varied; $w = [\sigma^2(F_o^2) + (0.0672P)^2 + 1.6197P]^{-1}$ where $P = [\mathrm{Max}(F_o^2, 0) + 2F_c^2]/3$).

$^g R_1 = \Sigma||F_o| - |F_c||/\Sigma|F_o|$; $wR_2 = [\Sigma w(F_o^2 - F_c^2)^2/\Sigma w(F_o^4)]^{1/2}$.

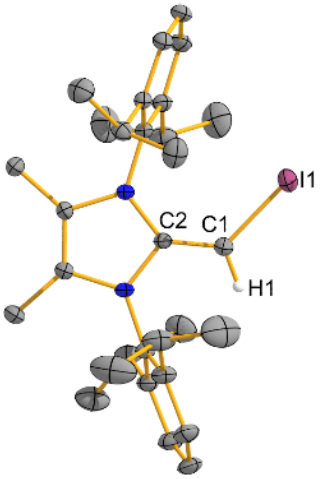

Figure S25. Molecular structure of ^{Me}IPr=CH(I) (**1**) plotted at a 30 % probability level. All hydrogen atoms (except the vinylic hydrogen) were omitted for clarity. Selected bond lengths [Å] and angles [°]: C1-I1 2.068(3), C2-C1 1.360(5); C2-C1-I1 128.1(3).

Table S2. Crystallographic details for $[(^{Me}IPrCH)Li]_2{\bullet}C_6H_{14}$ (**2**).

A. Crystal Data	
formula	$C_{66}H_{96}Li_2N_4$
formula weight	959.34
crystal dimensions (mm)	0.33 × 0.32 × 0.14
crystal system	monoclinic
space group	$P2_1/n$ (an alternate setting of $P2_1/c$ [No. 14])
unit cell parameters[a]	
a (Å)	12.287(4)
b (Å)	20.074(7)
c (Å)	12.726(4)
β (deg)	103.325(4)
V (Å^3)	3054.4(18)
Z	2
ρ_{calcd} (g cm^{-3})	1.043
μ (mm^{-1})	0.059
B. Data Collection and Refinement Conditions	
diffractometer	Bruker PLATFORM/APEX II CCD[b]
radiation (λ [Å])	graphite-monochromated Mo Kα (0.71073)
temperature (°C)	–80
scan type	ω scans (0.3°) (45 s exposures)
data collection 2θ limit (deg)	50.00
total data collected	20795 ($-14 \leq h \leq 14$, $-23 \leq k \leq 23$, $-15 \leq l \leq 15$)
independent reflections	5378 (R_{int} = 0.0511)
number of observed reflections (*NO*)	3431 [$F_o^2 \geq 2\sigma(F_o^2)$]
structure solution method	intrinsic phasing (*SHELXT-2014*[c])
refinement method	full-matrix least-squares on F^2 (*SHELXL–2016*[d])
absorption correction method	Gaussian integration (face-indexed)
range of transmission factors	1.0000–0.9275
data/restraints/parameters	5378 / 0 / 331
goodness-of-fit (S)[e] [all data]	1.019
final R indices[f]	
R_1 [$F_o^2 \geq 2\sigma(F_o^2)$]	0.0537
wR_2 [all data]	0.1671
largest difference peak and hole	0.349 and –0.221 e Å^{-3}

[a]Obtained from least-squares refinement of 4410 reflections with 4.62° < 2θ < 41.66°.

[b]Programs for diffractometer operation, data collection, data reduction and absorption correction were those supplied by Bruker.

[c]Sheldrick, G. M. *Acta Crystallogr.* 2015, **A71**, 3–8. (*SHELXT-2014*)

[d]Sheldrick, G. M. *Acta Crystallogr.* 2015, **C71**, 3–8. (*SHELXL-2016*)

[f]$S = [\Sigma w(F_o^2 - F_c^2)^2/(n - p)]^{1/2}$ (n = number of data; p = number of parameters varied; $w = [\sigma^2(F_o^2) + (0.0803P)^2 + 0.7570P]^{-1}$ where $P = [\mathrm{Max}(F_o^2, 0) + 2F_c^2]/3$).

[g]$R_1 = \Sigma||F_o| - |F_c||/\Sigma|F_o|$; $wR_2 = [\Sigma w(F_o^2 - F_c^2)^2/\Sigma w(F_o^4)]^{1/2}$.

Table S3. Crystallographic details for (^{Me}IPrCH)$_2$Sn•C_6H_{14} (**4**).

A. Crystal Data	
formula	$C_{66}H_{96}N_4Sn$
formula weight	1064.15
crystal dimensions (mm)	0.28 × 0.21 × 0.20
crystal system	monoclinic
space group	$I2/m$ (an alternate setting of $C2/m$ [No. 12])
unit cell parameters^a	
a (Å)	12.0876(3)
b (Å)	20.5096(6)
c (Å)	12.7329(3)
β (deg)	99.9583(9)
V (Å^3)	3109.08(14)
Z	2
ρ_{calcd} (g cm^{-3})	1.137
μ (mm^{-1})	3.557
B. Data Collection and Refinement Conditions	
diffractometer	Bruker D8/APEX II CCD^b
radiation (λ [Å])	Cu Kα (1.54178) (microfocus source)
temperature (°C)	–100
scan type	ω and ϕ scans (1.0°) (5 s exposures)
data collection 2θ limit (deg)	145.03
total data collected	10892 ($-14 \leq h \leq 14$, $-25 \leq k \leq 25$, $-15 \leq l \leq 15$)
independent reflections	3166 (R_{int} = 0.0182)
number of observed reflections (*NO*)	3165 [$F_o^2 \geq 2\sigma(F_o^2)$]
structure solution method	intrinsic phasing (*SHELXT-2014*^{c})
refinement method	full-matrix least-squares on F^2 (*SHELXL–2017*^{d})
absorption correction method	Gaussian integration (face-indexed)
range of transmission factors	0.6348–0.4873
data/restraints/parameters	3166 / 0 / 174
goodness-of-fit (S)^e[all data]	1.387
final R indices^f	
R_1 [$F_o^2 \geq 2\sigma(F_o^2)$]	0.0477
wR_2 [all data]	0.1122
largest difference peak and hole	0.227 and –0.828 e Å^{-3}

^{a}Obtained from least-squares refinement of 9449 reflections with 9.32° < 2θ <

144.60°.

[b]Programs for diffractometer operation, data collection, data reduction and absorption correction were those supplied by Bruker.

[c]Sheldrick, G. M. *Acta Crystallogr.* 2015, **A71**, 3–8. (*SHELXT-2014*)

[d]Sheldrick, G. M. *Acta Crystallogr.* 2015, **C71**, 3–8. (*SHELXL-2017*)

[e]$S = [\Sigma w(F_o^2 - F_c^2)^2/(n - p)]^{1/2}$ (n = number of data; p = number of parameters varied; $w = [\sigma^2(F_o^2) + (0.0058P)^2 + 8.5990P]^{-1}$ where $P = [\mathrm{Max}(F_o^2, 0) + 2F_c^2]/3$).

[f]$R_1 = \Sigma||F_o| - |F_c||/\Sigma|F_o|$; $wR_2 = [\Sigma w(F_o^2 - F_c^2)^2/\Sigma w(F_o^4)]^{1/2}$.

Table S4. Crystallographic details for **4′**.

A. Crystal Data	
formula	$C_{60}H_{82}N_4Sn$
formula weight	977.98
crystal dimensions (mm)	0.12 × 0.10 × 0.03
crystal system	monoclinic
space group	$P2_1/n$ (an alternate setting of $P2_1/c$ [No. 14])
unit cell parameters[a]	
a (Å)	10.683(2)
b (Å)	36.042(7)
c (Å)	14.412(3)
β (deg)	90.658(3)
V (Å^3)	5548.6(19)
Z	4
ρ_{calcd} (g cm^{-3})	1.171
μ (mm^{-1})	0.500
B. Data Collection and Refinement Conditions	
diffractometer	Bruker PLATFORM/APEX II CCD[b]
radiation (λ [Å])	graphite-monochromated Mo Kα (0.71073)
temperature (°C)	–80
scan type	ω scans (0.3°) (30 s exposures)
data collection 2θ limit (deg)	50.50
total data collected	38142 ($-12 \le h \le 12$, $-43 \le k \le 43$, $-17 \le l \le 17$)
independent reflections	10052 (R_{int} = 0.1240)
number of observed reflections (*NO*)	5180 [$F_o^2 \ge 2\sigma(F_o^2)$]
structure solution method	intrinsic phasing (*SHELXT-2014*[c])
refinement method	full-matrix least-squares on F^2 (*SHELXL–2016*[d])
absorption correction method	Gaussian integration (face-indexed)
range of transmission factors	1.0000–0.8890
data/restraints/parameters	10552 / 22[e] / 620
goodness-of-fit (S)[f] [all data]	0.998
final R indices[g]	
R_1 [$F_o^2 \ge 2\sigma(F_o^2)$]	0.0603
wR_2 [all data]	0.1847
largest difference peak and hole	0.999 and –0.745 e Å^{-3}

[a]Obtained from least-squares refinement of 2594 reflections with $4.42° < 2\theta < 33.44°$.

[b]Programs for diffractometer operation, data collection, data reduction and absorption correction were those supplied by Bruker.

[c]Sheldrick, G. M. *Acta Crystallogr.* 2015, **A71**, 3–8. (*SHELXT-2014*)

[d]Sheldrick, G. M. *Acta Crystallogr.* 2015, **C71**, 3–8. (*SHELXL-2016*)

[e]The C–C distances of the disordered isopropyl group (including the $\underline{C}_{aryl}$–$\underline{C}HMe_2$ distance) were restrained by use of the *SHELXL* **SADI** instruction. Additionally, a rigid-bond restraint (**RIGU**) was applied to the disordered isopropyl group. Finally, an anti-bumping distance restraint was applied to hydrogen atoms H6 and H42D to ensure a more reasonable orientation for the hydrogen atoms of methyl groups C6 and C42A.

[f]$S = [\Sigma w(F_o^2 - F_c^2)^2/(n - p)]^{1/2}$ (n = number of data; p = number of parameters varied; $w = [\sigma^2(F_o^2) + (0.0821P)^2]^{-1}$ where $P = [\mathrm{Max}(F_o^2, 0) + 2F_c^2]/3$).

[g]$R_1 = \Sigma||F_o| - |F_c||/\Sigma|F_o|$; $wR_2 = [\Sigma w(F_o^2 - F_c^2)^2/\Sigma w(F_o^4)]^{1/2}$.

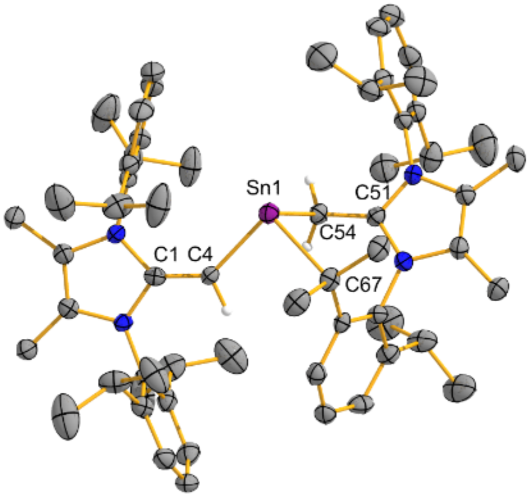

Figure S26. Molecular structure of **4′** with thermal ellipsoids plotted at a 30 % probability level. All hydrogen atoms (except the vinylic/olefinic hydrogen atoms) were omitted for clarity. Selected bond lengths [Å] and angles [°]: Sn1-C4 2.168(6), Sn1-C54 2.428(5), Sn1-C67 2.358(6), C1-C4 1.355(7), C51-C54 1.418(7); C4-Sn1-C54 94.5(2), C4-Sn1-C67 92.47(19), C54-Sn1-C67 89.82(19), Sn1-C54-C51 110.2(4), Sn1-C4-C1 135.0(4).

Table S5. Crystallographic details for (^{Me}IPrCH)$_2$Pb•C_6H_{14} (**5**).

A. Crystal Data	
formula	$C_{66}H_{96}N_4Pb$
formula weight	1151.64
crystal dimensions (mm)	0.29 × 0.26 ×0.19
crystal system	monoclinic
space group	$I2/m$ (an alternate setting of $C2/m$ [No. 12])
unit cell parameters[a]	
a (Å)	12.0808(3)
b (Å)	20.5866(5)
c (Å)	12.7242(3)
β (deg)	99.9820(10)
V (Å^3)	3116.64(13)
Z	2
ρ_{calcd} (g cm^{-3})	1.228
μ (mm^{-1})	5.550
B. Data Collection and Refinement Conditions	
diffractometer	Bruker D8/APEX II CCD[b]
radiation (λ [Å])	Cu Kα (1.54178) (microfocus source)
temperature (°C)	–100
scan type	ω and ϕ scans (1.0°) (5 s exposures)
data collection 2θ limit (deg)	147.89
total data collected	68854 ($-14 \leq h \leq 13$, $-25 \leq k \leq 25$, $-15 \leq l \leq 15$)
independent reflections	3216 (R_{int} = 0.0257)
number of observed reflections (NO)	3216 [$F_o^2 \geq 2\sigma(F_o^2)$]
structure solution method	intrinsic phasing (*SHELXT-2014*[c])
refinement method	full-matrix least-squares on F^2 (*SHELXL–2017*[d])
absorption correction method	Gaussian integration (face-indexed)
range of transmission factors	0.5199–0.3428
data/restraints/parameters	3216 / 0 / 175
goodness-of-fit (S)[e] [all data]	1.409
final R indices[f]	
R_1 [$F_o^2 \geq 2\sigma(F_o^2)$]	0.0446
wR_2 [all data]	0.1162
largest difference peak and hole	1.016 and –2.162 e Å^{-3}

[a]Obtained from least-squares refinement of 9858 reflections with 12.68° < 2θ < 147.52°.

[b]Programs for diffractometer operation, data collection, data reduction and absorption correction were those supplied by Bruker.

[c]Sheldrick, G. M. *Acta Crystallogr.* 2015, **A71**, 3–8. (*SHELXT-2014*)

[d]Sheldrick, G. M. *Acta Crystallogr.* 2015, **C71**, 3–8. (*SHELXL-2016*)

[e]$S = [\Sigma w(F_o^2 - F_c^2)^2/(n - p)]^{1/2}$ (n = number of data; p = number of parameters varied; $w = [\sigma^2(F_o^2) + 19.5228P]^{-1}$ where $P = [\mathrm{Max}(F_o^2, 0) + 2F_c^2]/3$).

[f]$R_1 = \Sigma||F_o| - |F_c||/\Sigma|F_o|$; $wR_2 = [\Sigma w(F_o^2 - F_c^2)^2/\Sigma w(F_o^4)]^{1/2}$.

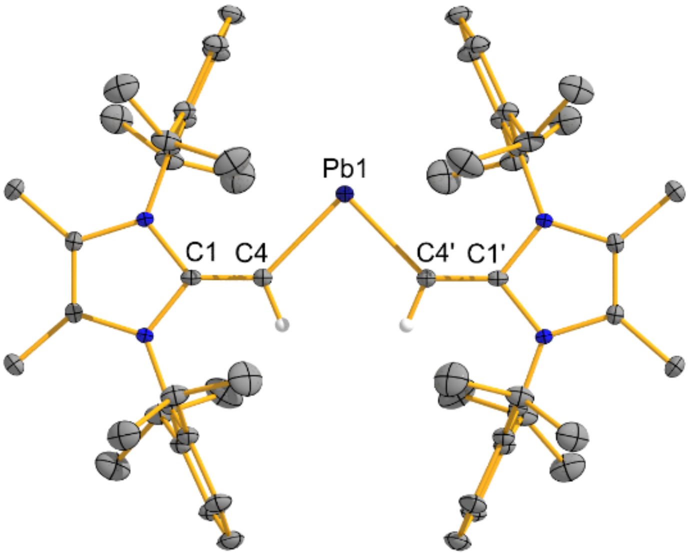

Figure S27. Molecular structure of (^{Me}IPrCH)$_2$Pb: (**5**) with thermal ellipsoids plotted at a 30 % probability level. All hydrogen atoms (except the vinylic hydrogens) were omitted for clarity. Selected bond lengths [Å] and angles [°]: C4-Pb1 2.210(4), C1-C4 1.342(7); C1-C4-Pb1 134.30(10), C4-Pb1-C4′ 88.6(2).

Table S6. Crystallographic details for $(^{Me}IPrCH)_2Si{\bullet}C_6H_{14}$ (**7**).

A. Crystal Data	
formula	$C_{66}H_{96}N_4Si$
formula weight	973.55
crystal dimensions (mm)	0.28 × 0.12 × 0.03
crystal system	monoclinic
space group	*I*2/*m* (an alternate setting of *C*2/*m* [No. 12])
unit cell parameters[a]	
a (Å)	12.1119(2)
b (Å)	20.0684(3)
c (Å)	12.7422(2)
β (deg)	99.7980(9)
V (Å^3)	3052.02(8)
Z	2
ρ_{calcd} (g cm^{-3})	1.059
μ (mm^{-1})	0.633
B. Data Collection and Refinement Conditions	
diffractometer	Bruker D8/APEX II CCD[b]
radiation (λ [Å])	Cu Kα (1.54178) (microfocus source)
temperature (°C)	–100
scan type	ω and ϕ scans (1.0°) (5 s exposures)
data collection 2θ limit (deg)	148.30
total data collected	10850 ($-15 \leq h \leq 15$, $-24 \leq k \leq 24$, $-15 \leq l \leq 14$)
independent reflections	3086 (R_{int} = 0.0267)
number of observed reflections (*NO*)	2771 [$F_o^2 \geq 2\sigma(F_o^2)$]
structure solution method	intrinsic phasing (*SHELXT-2014*[c])
refinement method	full-matrix least-squares on F^2 (*SHELXL–2017*[d])
absorption correction method	Gaussian integration (face-indexed)
range of transmission factors	0.9424–0.7203
data/restraints/parameters	3086 / 0 / 179
goodness-of-fit (*S*)[e] [all data]	1.038
final *R* indices[f]	
R_1 [$F_o^2 \geq 2\sigma(F_o^2)$]	0.0413
wR_2 [all data]	0.1142
largest difference peak and hole	0.241 and –0.275 e Å^{-3}

[a]Obtained from least-squares refinement of 9874 reflections with 8.30° < 2θ <

148.06°.

[b]Programs for diffractometer operation, data collection, data reduction and absorption correction were those supplied by Bruker.

[c]Sheldrick, G. M. *Acta Crystallogr.* 2015, **A71**, 3–8. (*SHELXT-2014*)

[d]Sheldrick, G. M. *Acta Crystallogr.* 2015, **C71**, 3–8. (*SHELXL-2017*)

[e]$S = [\Sigma w(F_o^2 - F_c^2)^2/(n - p)]^{1/2}$ (n = number of data; p = number of parameters varied; $w = [\sigma^2(F_o^2) + (0.0571P)^2 + 1.6993P]^{-1}$ where $P = [\mathrm{Max}(F_o^2, 0) + 2F_c^2]/3$).

[f]$R_1 = \Sigma||F_o| - |F_c||/\Sigma|F_o|$; $wR_2 = [\Sigma w(F_o^2 - F_c^2)^2/\Sigma w(F_o^4)]^{1/2}$.

Table S7. Crystallographic details for (^{Me}IPrCH)$_2$Si(dba) (**8**).

A. Crystal Data	
formula	$C_{77}H_{96}N_4OSi$
formula weight	1121.66
crystal dimensions (mm)	0.17 × 0.08 × 0.04
crystal system	orthorhombic
space group	$P2_12_12_1$ (No. 19)
unit cell parameters^a	
a (Å)	12.6733(4)
b (Å)	22.4008(7)
c (Å)	23.4422(7)
V (Å^3)	6655.1(4)
Z	4
ρ_{calcd} (g cm^{-3})	1.119
μ (mm^{-1})	0.658
B. Data Collection and Refinement Conditions	
diffractometer	Bruker D8/APEX II CCD^b
radiation (λ [Å])	Cu Kα (1.54178) (microfocus source)
temperature (°C)	–100
scan type	ω and ϕ scans (1.0°) (5-10-15 s exposures)^c
data collection 2θ limit (deg)	142.02
total data collected	113027 ($-15 \le h \le 15$, $-27 \le k \le 26$, $-27 \le l \le 24$)
independent reflections	12607 (R_{int} = 0.0877)
number of observed reflections (NO)	9854 [$F_o^2 \ge 2\sigma(F_o^2)$]
structure solution method	intrinsic phasing (*SHELXT-2014*^{d})
refinement method	full-matrix least-squares on F^2 (*SHELXL–2018*^{e})
absorption correction method	Gaussian integration (face-indexed)
range of transmission factors	0.9795-0.8758
data/restraints/parameters	12607 / 36^f / 781
extinction coefficient (x)^g	0.00052(6)
Flack absolute structure parameter^h	-0.016(14)
goodness-of-fit (S)^i [all data]	1.119
final R indices^j	
R_1 [$F_o^2 \ge 2\sigma(F_o^2)$]	0.0533
wR_2 [all data]	0.1311
largest difference peak and hole	0.206 and –0.236 e Å^{-3}

^{a}Obtained from least-squares refinement of 9933 reflections with 5.46° < 2θ <

137.72°.

[b]Programs for diffractometer operation, data collection, data reduction and absorption correction were those supplied by Bruke

[c]Data were collected with the detector set at three different positions. Low-angle (detector 2θ = –33°) data frames were collected using a scan time of 5 s, medium-angle (detector 2θ = 75°) frames using a scan time of 10 s, and high-angle (detector 2θ = 117°) frames using a scan time of 15 s.

[d]Sheldrick, G. M. *Acta Crystallogr.* 2015, **A71**, 3–8. (*SHELXT-2014*)

[e]Sheldrick, G. M. *Acta Crystallogr.* 2015, **C71**, 3–8. (*SHELXL-2018/3*)

[f]The disordered isopropyl group had the following same distance restraints (**SADI**): C36–C40A & C36–C40B; C40A–C41A & C40B–C41B; C40A–C42A & C40B–C42B; C36···C41A & C36···C41A; C36···C42A & C36···C42B; C41A···C42A & C41A···C42A. Additionally, the rigid bond restraint (**RIGU**) was applied to the anisotropic displacement parameters of the carbon atoms of the disordered isopropyl group.

[g]$F_c^* = kF_c[1 + x\{0.001F_c^2\lambda^3/\sin(2\theta)\}]^{-1/4}$ where k is the overall scale factor.

[h]Flack, H. D. *Acta Crystallogr.* 1983, **A39**, 876–881; Flack, H. D.; Bernardinelli, G. *Acta Crystallogr.* 1999, **A55**, 908–915; Flack, H. D.; Bernardinelli, G. *J. Appl. Cryst.* 2000, **33**, 1143–1148. The Flack parameter will refine to a value near zero if the structure is in the correct configuration and will refine to a value near one for the inverted configuration.

[i]$S = [\Sigma w(F_o^2 - F_c^2)^2/(n - p)]^{1/2}$ (n = number of data; p = number of parameters varied; $w = [\sigma^2(F_o^2) + (0.0411P)^2 + 2.5833P]^{-1}$ where $P = [\mathrm{Max}(F_o^2, 0) + 2F_c^2]/3$).

[j]$R_1 = \Sigma||F_o| - |F_c||/\Sigma|F_o|$; $wR_2 = [\Sigma w(F_o^2 - F_c^2)^2/\Sigma w(F_o^4)]^{1/2}$.

Cyclic Voltammetry Data

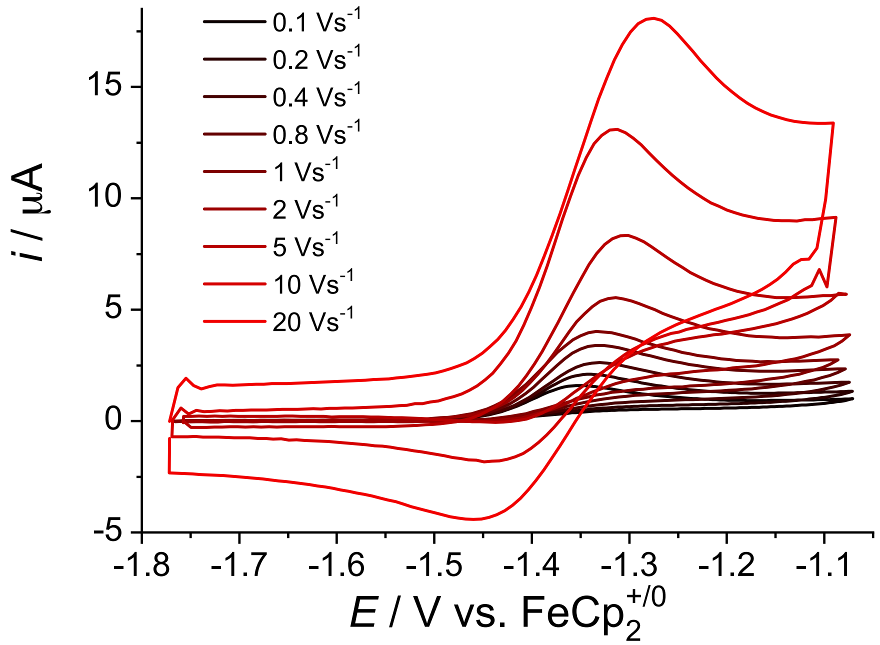

Figure S28. Scan rate dependent CV data of **7** (2 mM in benzene) under N_2 atmosphere, 0.2 M $^{n}Hex_4N[B(C_6F_5)_4]$ as an electrolyte.

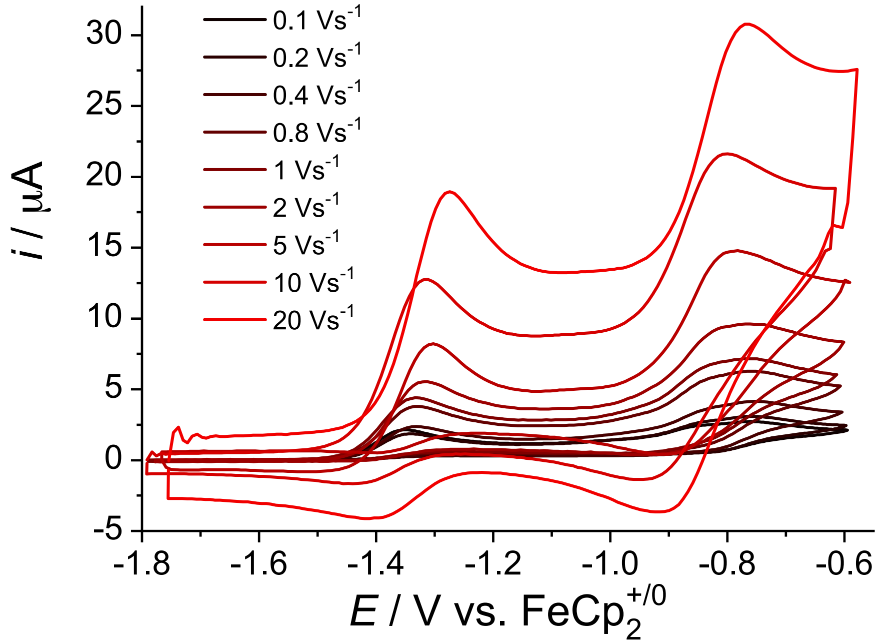

Figure S29. Scan rate dependent CV data of **7** (2 mM in benzene) under N_2 atmosphere, 0.2 M $^{n}Hex_4N[B(C_6F_5)_4]$ as an electrolyte.

UV-Visible Spectra and TD-DFT Assignments

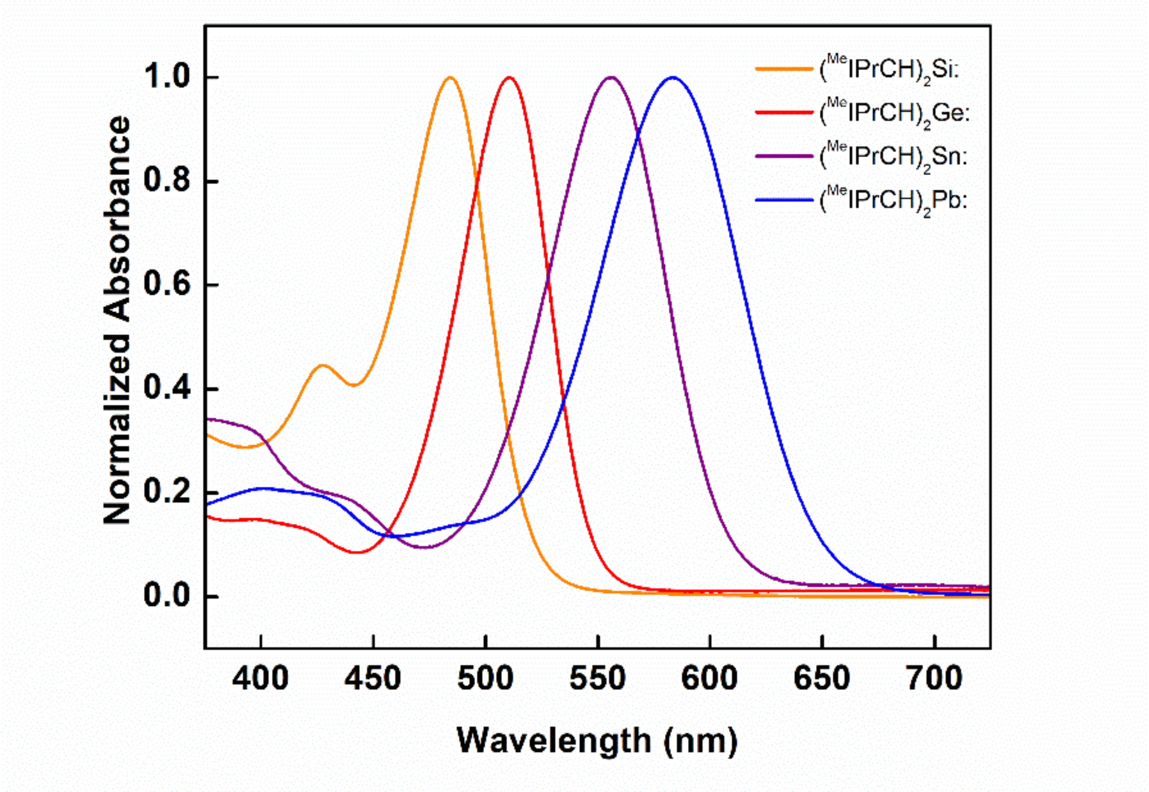

Figure S30. Experimentally determined UV-Visible spectra of divinyltetrelenes (^{Me}IPrCH)$_2$E: (E = Si–Pb) measured in hexanes.

Table S8. TD-DFT assigned dominant electronic transitions of divinyltetrelenes $(^{Me}IPrCH)_2E$: (E = Si–Pb).

Compound	λ_{max} (exp.) [nm]	λ_{max} (calcd.) [nm][a]	f[a]	Molecular orbital contributions[a]
$(^{Me}IPrCH)_2Si$:	428	421	0.1526	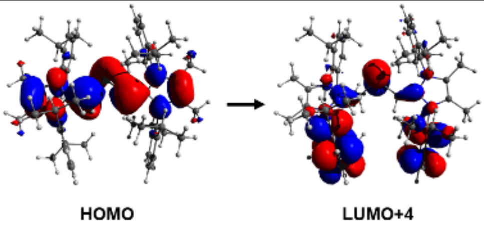
	484	465	0.2065	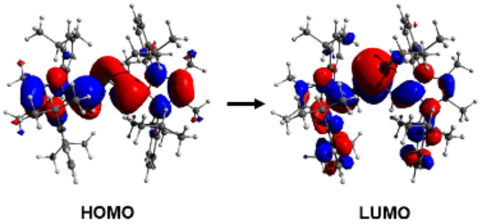
$(^{Me}IPrCH)_2Ge$:	511	480	0.4021	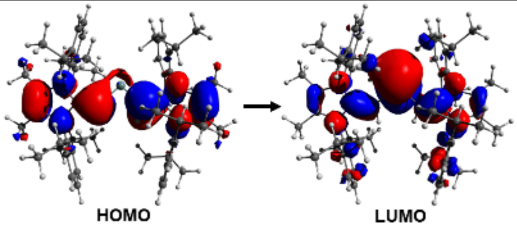
$(^{Me}IPrCH)_2Sn$:	557	534	0.3713	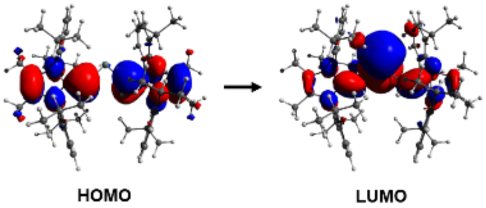
$(^{Me}IPrCH)_2Pb$:	583	571	0.3427	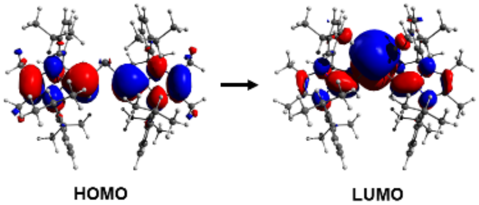

[a]TD-DFT calculated at the B3LYP/cc-pVDZ level of theory

Table S9. Selected computational data for divinyltetrelenes (^{Me}IPrCH)$_2$E: (E = Si–Pb).

	**(^{Me}IPrCH)$_2$Si**	**(^{Me}IPrCH)$_2$Ge**	**(^{Me}IPrCH)$_2$Sn**	**(^{Me}IPrCH)$_2$Pb**
E_{HOMO} (kcal/mol)	−83.1	−82.2	−81.5	−79.9
E_{LUMO} (kcal/mol)	−9.75	−12.6	−18.4	−20.4
$\Delta E_{HOMO–LUMO}$ (kcal/mol)	73.3	69.6	63.2	59.5
$\Delta E_{singlet–triplet}$ (kcal/mol)	37.6	41.4	39.7	36.5
LP orbital character	$s^{0.71}p^{0.29}$	$s^{0.77}p^{0.23}$	$s^{0.84}p^{0.16}$	$s^{0.90}p^{0.10}$
^{Me}IPr***C*=*C*** Wiberg bond index	1.50	1.52	1.56	1.59
C*–*E Wiberg bond index	0.93	0.93	0.83	0.81
C–E Natural Charge	−0.97	−0.93	−0.96	−0.93
C–***E*** Natural Charge	+0.70	+0.68	+0.81	+0.82
